# The intersection between menopause and depression: overview of research using animal models

**DOI:** 10.3389/fpsyt.2024.1408878

**Published:** 2024-07-15

**Authors:** José Jaime Herrera-Pérez, Olivia Tania Hernández-Hernández, Mónica Flores-Ramos, Jonathan Cueto-Escobedo, Juan Francisco Rodríguez-Landa, Lucía Martínez-Mota

**Affiliations:** ^1^ Laboratorio de Farmacología Conductual, Dirección de Investigaciones en Neurociencias, Instituto Nacional de Psiquiatría Ramón de la Fuente Muñiz, Mexico City, Mexico; ^2^ Consejo Nacional de Humanidades, Ciencias y Tecnologías Research Fellow. Instituto Nacional de Psiquiatría Ramón de la Fuente Muñiz, Mexico City, Mexico; ^3^ Subdirección de Investigaciones Clínicas, Instituto Nacional de Psiquiatría Ramón de la Fuente Muñiz, Mexico City, Mexico; ^4^ Departamento de Investigación Clínica, Instituto de Ciencias de la Salud, Universidad Veracruzana, Xalapa-Enríquez, Mexico; ^5^ Laboratorio de Neurofarmacología, Instituto de Neuroetología, Universidad Veracruzana, Xalapa-Enríquez, Mexico

**Keywords:** aging, perimenopause, estropause, ovarian hormones, accelerated ovarian failure, middle age, ovariectomy, post-ovariectomy time frame

## Abstract

Menopausal women may experience symptoms of depression, sometimes even progressing clinical depression requiring treatment to improve quality of life. While varying levels of estrogen in perimenopause may contribute to an increased biological vulnerability to mood disturbances, the effectiveness of estrogen replacement therapy (ERT) in the relief of depressive symptoms remains controversial. Menopausal depression has a complex, multifactorial etiology, that has limited the identification of optimal treatment strategies for the management of this psychiatric complaint. Nevertheless, clinical evidence increasingly supports the notion that estrogen exerts neuroprotective effects on brain structures related to mood regulation. Indeed, research using preclinical animal models continues to improve our understanding of menopause and the effectiveness of ERT and other substances at treating depression-like behaviors. However, questions regarding the efficacy of ERT in perimenopause have been raised. These questions may be answered by further investigation using specific animal models of reduced ovarian function. This review compares and discusses the advantages and pitfalls of different models emulating the menopausal stages and their relationship with the onset of depressive-like signs, as well as the efficacy and mechanisms of conventional and novel ERTs in treating depressive-like behavior. Ovariectomized young rats, middle-to-old aged intact rats, and females treated with reprotoxics have all been used as models of menopause, with stages ranging from surgical menopause to perimenopause. Additionally, this manuscript discusses the impact of organistic and therapeutic variables that may improve or reduce the antidepressant response of females to ERT. Findings from these models have revealed the complexity of the dynamic changes occurring in brain function during menopausal transition, reinforcing the idea that the best approach is timely intervention considering the opportunity window, in addition to the careful selection of treatment according to the presence or absence of reproductive tissue. Additionally, data from animal models has yielded evidence to support new promising estrogens that could be considered as ERTs with antidepressant properties and actions in endocrine situations in which traditional ERTs are not effective.

## 1 Introduction

There is significant epidemiological and clinical data to unequivocally support the notion that women experience more psychiatric problems, particularly mood and anxiety symptoms and sleep disorders, at some point in their lives than men (1–3). It is also known that for some women, this increased risk might be associated with reproductive events, such as the premenstrual or postpartum periods (4, 5), in which gonadal hormone levels drastically decrease, resulting in consequences on neuronal excitability, brain metabolism, and corticolimbic processing. The menopausal transition, also termed perimenopause, is defined as the phase in middle-age during which a woman transitions from a reproductive to a non-reproductive period. Perimenopause is characterized by sudden increases and decreases in estrogen levels linked to the aging of ovaries, and the transition to a non-reproductive life; this imbalance in the hormone milieu produces intense symptoms such as hot flushes, insomnia, irritability, and low libido (6, 7). Fluctuations in estrogen levels during perimenopause have also been linked to symptoms of depression, anxiety, and mild cognitive complaints, which are particularly relevant in a subset of middle-aged women with high sensitivity to extreme changes in estrogen levels, as they might suffer from psychiatric disorders that require treatment (4, 8, 9).

Natural menopause, that is, the last menstrual cycle, occurs between 45-53 years in most women. At this age, women transition out of the reproductive stage due to a drastic loss of ovulatory function and cessation of menses. As antral ovarian follicles are reduced, the levels of follicle-stimulating hormone (FSH) rise, denoting the beginning of the menopausal transition. A group of experts from the Stages of Reproductive Ageing Workshop *(*STRAW) proposed the menstruation pattern as a better indicator of the peri- and menopause stages, defining three stages as follows: a) the early transition to menopause characterized by the persistence of an irregular menstrual cycle, b) the late transition to menopause characterized by a period of amenorrhea greater than 60 days in the last year, and c) early post-menopause, defined as the interval of 12-24 months after the last menstruation (10). In terms of endocrine aging, FSH increases in early perimenopause and post-menopause with fluctuating levels; this hormone peaks before the last menstruation and is associated with late perimenopause. Perturbations in FSH release result in erratic levels of estradiol (E_2,_ the most abundant and potent estrogen produced by ovaries) and progesterone, which gradually decline until relatively low and stable concentrations are achieved (10). Notably, rapid fluctuations in E_2_ levels may have implications for women’s mental health, influencing factors such as the presence of depressive symptoms during the transition to menopause (4).

The endocrine and neurobiological bases that underlie the physiological and neuropsychiatric symptoms associated with the transition to menopause have been investigated in animal models that mimic the long-term effects of the absence of ovarian hormones. Several aspects of natural and surgical menopause appear to be similar between humans and other mammalian species; however, other important characteristics may be species-specific, thus limiting translational research (11). However, humans and model animals show general similarities in hormone profiles across menopausal transitions, with both showing the termination of irregular cycles, declining fertility with age, changes in low- and high-density lipoprotein cholesterol, a decline in serum dehydroepiandrosterone, and alterations in temperature-regulation systems (12). In addition, animal models respond adequately to the beneficial effects of estrogen replacement therapy (ERT), showed improvements in the alterations of bone metabolism, lipid profiles, and cognitive changes. Interestingly, in the long term, an increase in irritability, cognitive alterations, deterioration of memory processes, as well as the onset of anxiety- and depression-like behaviors have been observed in model animals, all of which support their use in research to understand the impact of the long-term absence of steroid hormones in several organs, including the brain (13, 14). Thus, the use of animal models can help to elucidate the mechanisms involved in diseases and alterations during human menopause, contributing to solving health problems at this physiological stage in women. Additionally, these models allow us to understand the physiological, endocrine, and neural mechanisms associated with the menopausal transition, and to evaluate potential therapeutic strategies to restore such alterations, which could contribute to improving the quality of life of this group of women (15–17).

### 1.1 Menopause and depression

Depression is the primary cause of the disability in women worldwide producing a significant impairment of their normal functioning and quality of life (1). Global studies have reported that prevalence of depression in menopause women is 35.6% (95% CI: 32.0–39.2%), ranging from 33.9% (95% CI: 27.8–40.0%) in perimenopausal women to 34.9% (95% CI: 30.7–39.1%) in postmenopausal women. These results come from studies with a high degree of heterogeneity, highlighting that higher rates of depression have been detected in the studies with poorer quality and smaller sample size, which is a limitation for interpretation of the findings (18). While multicentric cross-sectional studies have shown contradictory results regarding the relationship between menopausal status and higher rates of depression, longitudinal studies in women aged 40-55 years old have identified relationships between the naturally occurring endocrine changes in the menopausal transition with mood disturbances. Increased vulnerability to depressive symptoms has previously been reported in perimenopause with odds ratios ranging from 1.33 to 1.79 in women with a history of symptoms; this observation was replicated in women who previously had mood complaints (19, 20), suggesting that menopause may reduce the ability of women to cope with stressors, leading them to experience a higher depression symptom burden. Interestingly, structured evaluations applied to detect depression as a syndrome in a small group of premenopausal women found that the rate of depression doubled when women reached perimenopause, and tripled in postmenopause, indicating that subsets of women may suffer from more complex forms of depressive symptoms (8, 21). Additionally, surgical menopause elicited by oophorectomy has been associated with a higher risk of depression (22–24), as well as augmented levels of anxiety (25), which are linked to an abrupt reduction in sex steroid levels. Different studies have found that the patient’s age at the time of surgery, the type of surgery (uni- or bilateral), and additional extirpation of the uterus all modulate the risk of depressive symptoms. Thus, women who were younger at the time of surgery (25), in addition to women older than 51 years (26), were both found to exhibit a higher risk of affective symptoms, while an increased risk for depression (syndromic) and complaints in general health are registered in women subjected to the extirpation of the uterus and ovaries (27).

Symptoms of the climacteric period that are common among menopausal women may contribute to discomfort and recurrence of depressive symptoms (28, 29), such as hot flashes (found at a rate of 60 to 89%) (5), or sleep alterations (experienced by 23 to 42% of menopausal women) (30, 31). Psychological features in patients, such as anxiety traits and neuroticism, may also contribute to an increased vulnerability to depressive symptoms during perimenopause (32), suggesting an interaction between psychological and biological variables in resilience or vulnerability to developing depressive symptoms during menopause.

#### 1.1.1 Hypotheses to explain depression symptoms or mood disorders in menopause

Several hypotheses have been proposed to explain the increase in depression symptoms or mood disorders in menopause. At the neurological level, peri- and post-menopause have both been associated with lower gray matter volume in several cortical regions and subcortical structures (such as the hippocampus, amygdala, and thalamus) that regulate emotions, concurrent with a widespread loss of white matter in the major tracts interconnecting the cerebral cortex and subcortical regions, as well as hypometabolism in the parieto-temporal cortices (33). Volumetric changes were further reported in a meta-analytic review in which post-menopausal women (age 50-70 years old) had lower hippocampal and amygdala volumes relative to premenopausal women or men (34).

Variations in estrogen levels across perimenopause have been considered as factors influencing the etiology of mood symptoms (35). Estrogens play critical roles in modulating brain homeostasis, neural plasticity, and neuroprotection. In the model of classic actions of estrogens, these steroids were found to bind to the estrogen cytoplasmic receptors of subtypes α (ER-α) and β (ER-β), which are transported to the cell nucleus to stimulate transcription, producing slow effects in different tissues. However, estrogens also exert short-term effects mediated by non-traditional rapid/non-genomic/membrane-initiated estrogen signaling in different cell types, a mechanism mediated by calcium release and protein kinases (36). Estrogens have further been found to be involved in brain remodeling through the modulation spine dendritic sprouting in areas predominantly associated with cognition, anxiety, and mood, such as hippocampal formation, cerebral cortex, lateral septum and amygdala (37–39), among others. These steroids also participate in the synthesis, release, and metabolism of neurotransmitters such as monoamines, glutamate, acetylcholine, and GABA, and thus modulate neuronal excitability. As a neuroprotector, E_2_ has been proven to reduce NMDA excitotoxicity by influencing rapid actions mediated by membrane receptors, as well as by increasing the transcription of anti-apoptotic proteins by traditional intracellular mechanisms (40). These oscillations in the concentrations of steroids and neuropeptides impact serotonin and noradrenaline pathways which underlie mood disorders in woman in the menopausal transition or post-menopause; conversely, hormonal therapy in perimenopausal transition decreases the transport and catabolism of serotonin (41), and protects glucose metabolism in brain regions such as the hippocampus, entorhinal cortex, medial temporal cortex and posterior cingulate which have shown deterioration in women without hormonal therapy (42).

In support of the estrogen hypothesis, studies have reported that a longer duration of exposure to natural estrogens from the menarche to menopause transition was significantly associated with a reduced risk of depression in perimenopausal women, while a similar result was found in women with a history of prolonged use of oral contraceptives (43). Furthermore, women with a history of perimenopausal depression who were treated with E_2_ for three weeks, subsequently switching to placebo treatment, experienced an increase in the severity of depression symptoms; however, women with a history of depression that remains in the E_2_ arm of the study, as well as women without a history of depression in the arm with placebo, remained asymptomatic (44).

#### 1.1.2 Use of hormone replaced therapy to relieve depressive symptoms

In recent years, the life expectancy of the population has increased, while psychiatric symptoms have been found to greatly decrease the quality of life of women; as such, it is necessary to develop treatments that relieve depression. Women experiencing different health issues during menopause may favor estrogenic hormone replacement therapy (HRT); however, in terms of mood regulation or depression symptoms, double-blind clinical trials have provided limited evidence regarding the efficacy of HRT in reducing affective symptoms during menopause (45). However, HRT seems to be more effective at reducing depressive symptoms in women in the early transition to menopause than in those in the late transition (46), thus strengthening the notion of an opportunity window for therapeutic intervention. Evidence collected from prior clinical studies have shown that methodological differences in aspects such as the inclusion of participants with or without a depression diagnosis (versus women with depression symptoms), or conducting evaluations at different stages of perimenopause, may contribute to a positive HRT response. Considering that the peri- and post-menopause stages are associated with varying release of estrogens and gonadotrophin levels in such a period, and individuality in the response to hormones, it is not surprising to find heterogeneity in the results of HRT with estrogens on depression symptoms in menopause.

Traditional HRT may include either a single estrogen, a combination of estrogens, or a combination of estrogens plus a progestogen. This treatment has been recognized by some clinical guides as a useful and effective therapy for the control of depressive symptoms (with the limitations described above), or as an adjuvant in the management of depression with antidepressant drugs (47). New treatments for depression and anxiety symptoms during menopause include synthetic estrogens, phytoestrogens, and molecules derived from plants used in folk medicine, such as flavonoids. The primary assessment of the efficacy and safety of these therapeutic approaches, as well as the elucidation of their mechanisms of action to induce antidepressant-like effects, have predominantly been investigated in different animal models of menopause.

## 2 Animal models for the study of HRT and depression

In most mammalian species, females experience natural reproductive senescence in mid- to late-life; however, most species’ lifespans do not surpass their reproductive years. However, currently, human females live one-third of their lives after passing beyond the reproductive stage (48). Some aspects of reproductive senescence are not readily accessible to humans; thus, animal models are important tools for studying events occurring in human menopause at multiple levels, including the organ, system, cellular, molecular, and genomic levels. The endocrine and neuroendocrine aspects of these model should resemble those of human menopause to ensure translational validity. This will allow the knowledge found in animal models to be translated to humans, and ensures the relevancy of the study to the biological basis of human menopause and its therapeutic interventions, including the discovery of new agents for HRT (49).

The use of non-human primates as animal models of menopause is particularly useful because of their genetic, physiological, behavioral, and reproductive similarities to humans; however, these models are costly, and the study of reproductive senescence in this model may require many years due to their long lifespan. In this regard, rodents such as mice and rats have a short lifespan of two to three-years, making them useful models to study menopause, since rodent ovaries deteriorate with age. Rodent models have been used to evaluate the effects of gonadal hormones on various body systems, including developmental and cognitive processes (48). Furthermore, the neurobiological features of rodents make them excellent models for studying the expression of stress-evoked responses, depressive-like behaviors (i.e., despair and anhedonia), and other signs that often develop comorbid with depression, such as anxiety and alert responses.

**Figure 1:**
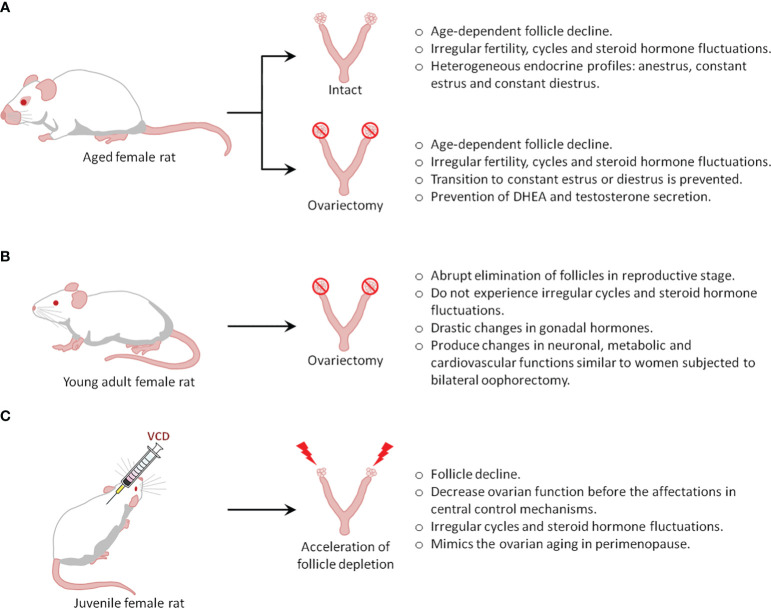
Types of menopause rat models.

There are 3 types of menopausal models: intact aging (natural menopause), surgical menopause or ovariectomy, and accelerated ovarian failure (**Figure 1**). The choice of the animal model generally depends on the study objectives.

### 2.1 The intact aging rat model (natural reproductive senescence)

Humans and rats both follow similar stages of reproductive senescence, transitioning from regular cycling to irregular cycling to acyclicity. Further, they share multiple features and endocrine changes, including a) a decline in follicles, b) irregular cycling and steroid hormone fluctuations, and c) irregular fertility (50). In rodents, the length of their estrous cycle starts to become irregular at 8 months of age, indicating reproductive senescence, while at the age of 21 months, rats do not show cyclic changes in serum E_2_, progesterone, luteinizing hormone (LH), and FSH (51). The reliability of these changes in laboratory rodents and similar hormonal fluctuations in middle-aged rodents and women provides a well-defined model of irregular cycles of human menopause (49).

One drawback of this model is that rodents do not present true menopause, which is characterized by low to undetectable estrogen levels. Instead, they undergo reproductive senescence, referred to as ‘estropause’, which is instead characterized by low but persistent estrogen levels. Indeed, most aging rodents (60-70%) show a spontaneous transition to a constant estrus, characterized by sustained levels of E_2_ and low levels of progesterone that can last 10-100 days (50, 51). Thus, aged animals (21 months) showed well-developed ovarian follicles, but not corpora lutea, and further show higher levels of hypothalamic GnRH, pituitary FSH, and prolactin compared to estrus in 3-month-old cycling rats. Furthermore, pituitary LH is significantly lower in old rats (52). The remaining 30-40% of aging rodents transition from irregular cycling to anestrus characterized by consistently low levels of E_2_, progesterone, LH, and FSH (50, 51, 53); as such, these animals may represent a better model of the human perimenopause to menopause transition. Although all rodents enter a state of anestrus eventually, the period of constant estrus and anestrus may induce substantial differences in the rat brain (49). Additionally, a pseudo-gestational state (permanent diestrus) has also been described in aging rodents, as the ovaries of these animals develop corpora lutea, which may be transitory, or last for the rest of the rodent’s lifespan. Endocrinologically, this stage is characterized by low E_2_ and FSH levels, but the considerable production of progesterone (51, 53), a hormone that also induces permanent changes in the brain of rodents.

Overall, it is clear that rodent aging is associated with heterogeneous endocrine profiles, the least predominant being a transition to anestrus, which better resembles the human perimenopause-to-menopause transition. To increase the number of animals in anestrus, one common strategy is to ovariectomize rodents at an age when irregular cycles begin to develop, which prevents the establishment of constant estrus and diestrus in aged animals. However, this strategy introduces another variable that would differentiate the model from human menopause, caused by the loss of the ovaries, which are known to secrete hormones, including testosterone and dehydroepiandrosterone (DHEA), in menopausal women (54, 55).

#### 2.1.1 Differences between human and rodent senescence

Differences in reproductive senescence between humans and rodents have been determined based on neuroendocrine dissimilarities (**Table 1**). As women age, follicular reserves and serum estrogen and progesterone levels gradually decrease. In contrast, in aging rats, as their ovaries retain follicles throughout their lifespan, their estrogen levels do not decrease to undetectable levels; thus, the persistent estrus state is caused by a slightly elevated estrogen level, while the high progesterone levels in persistent diestrus occur secondary to increased corpora lutea activity (53). These data support the idea that the primary mechanism that ultimately results in reproductive senescence in women is ovarian follicle depletion, whereas in rats, it is an alteration in the hypothalamic-pituitary-gonadal (HPG) axis (9).

**Table 1:** Key differences between rodent and human reproductive senescence.

	Women	Female rats
**Reproductive stage**	Menstrual 28 days cycle.	Estrous 4-5 days cycle.
	Three phases/cycle: Follicular, periovulatory and luteal.	Four phases/cycle: Metestrus, diestrus, proestrus and estrus.
	Similar ovarian hormone fluctuations.	
	Uterine lining is shed via menstruation.	Uterine lining is reabsorbed.
**Reproductive senescence**	Appears at 45 – 55 years of age.	Appears at 9 – 12 months of age.
	Irregular cycles.	
	Decline in follicles.	
	Irregular ovarian hormone fluctuations.	
	*Menopause*: low to undetectable levels of estradiol and progesterona, increased levels of LH and FSH.	*Estropause(persistent estrus state)*: moderate to high estradiol levels, and moderate progesterone, LH and FSH levels.
	Determined by complete ovarian failure.	Determined by deregulated Hypothalamus-Pituitary-Gonadal axis

Although the basic mechanism responsible for rodent reproductive senescence differs from that of human menopause, the aging rodent model can still be used to provide insights into the general mechanisms or consequences of menopause.

### 2.2 Studies based on aged rat as a menopause model for the study of depression

Aged rats may be an ideal menopausal model to study the role of ovarian hormones in the pathogenesis of depression and the mechanisms underlying the antidepressant efficacy of drugs and hormones. However, intact aged rats have been scarcely used for this purpose because they show complex endocrine regulation, resulting in the development several types of endocrine profiles, as stated above. Thus, to prevent these different profiles, ovariectomized aged rats are more frequently used.

#### 2.2.1 Studies based on gonadally-intact aged female rat

The few published studies using gonadally intact aged females have provided limited information regarding the relationship between ovarian hormones, menopause, and depression symptoms; instead, these animals have been used to study the novel mechanisms of anxiolytic or antidepressant actions of drugs or E_2_. For example, one study showed that anxiety and depression behavior in aged females (26 m.o.) were reduced by treatment with a G protein-coupled estrogen receptor (GPER) agonist (G-1) which also could increase intracellular ER-α and ER-β (56). Additionally, one study evaluated the anxiolytic and antidepressant effects of Cannabidiol in middle-aged intact rats (13 months old) exposed to social isolation, finding that this drug reduced depressive and anxiety behaviors induced by the stressor (57). Although corticosterone has typically been associated with depressive behaviors, recent data have suggested a putative antidepressant effect of corticosterone (58). In this study, corticosterone was found to exert mild antidepressant effects in aged intact female rats. These data indicate an improvement in anxiolytic and antidepressant behaviors; however, the mechanisms are not directly related to endocrine changes associated with menopause.

In contrast to the anxiolytic and antidepressant effects observed in intact aged rats, one study found no effects of E_2_ and prolame (a synthetic estrogen) on anxious or depressive behavior in gonadally- intact female rats coursing with irregular estrous cycles (14 months); however, when the aged rats were ovariectomized, prolame exerted anxiolytic and antidepressant actions (59). Thus, to better understand the role of these hormones in depression and its treatment, it would be useful to compare the endocrine statuses of all animals. In this regard, one study evaluating the influence of age (reproductive senescence) on the efficacy of fluoxetine in young, middle-aged, and senescent rats in the metestrus/diestrus phase of their estrous cycle found that as female rats aged, the antidepressant effect of fluoxetine was reduced (60), suggesting that age-related endocrine changes may reduce the efficacy of fluoxetine to alleviate depressive behavior. Classic neuroendocrine studies have demonstrated that the transition from the reproductive to non-reproductive stages in rats alters the diurnal rhythm of neurotransmitter release from the mesencephalic nuclei (61, 62), suggesting that the estropause transition is associated with a generalized alteration in neurotransmitter systems, such as those relevant to the actions of antidepressant drugs.

#### 2.2.2 Studies in ovariectomized aged female rats

Considering the endocrine complexity of aging females, ovariectomized aged rats are most frequently used to study the role of gonadal hormones in depressive-like behaviors, while several hypotheses regarding the relationship between hormones (mainly E_2_), depression, and the perimenopause-to-menopause transition have been tested.

##### 2.2.2.1 Ovariectomy induces depressive-like behaviors in aged rats

To examine the effects of long-term ovarian hormone deprivation on the development of depressive-like behaviors, Sprague-Dawley rats were ovariectomized at 5 months of age, four months later they were subjected to 6 weeks of chronic unpredictable stress and tested in the forced swimming test (FST, an animal model of depression) at 10 months of age. Overall, this treatment increased depressive-like behavior in the FST and also induced an anhedonic state. Long-term ovariectomy impaired the negative feedback of the hypothalamus-pituitary-adrenal (HPA) axis (57). These results suggest that ovarian hormones are relevant for assessing the resilience to chronic unpredictable stress in aged female rats, and that E_2_ restitution in ovariectomized aged rats may confer resilience to stress and reduce depressive- or anxiety-like behaviors.

##### 2.2.2.2 E_2_ produces antidepressant-like effects in aged rats

Prior research has shown that chronic E_2_ treatment (over 10 weeks), as well as vitamin D treatment, induces antidepressant effects in ovariectomized middle-aged rats (12 months); both compounds upregulate each other’s receptors and show neuroprotective effects (63). In the chronic mild stress paradigm, an animal model of anhedonia, a single injection of estradiol valerate produced antidepressant-like actions in ovariectomized middle-aged rats evaluated 3 weeks after surgery (64). It has also been observed that ethinyl-estradiol (a synthetic analog of E_2_) induces an antidepressant effect in middle-aged rats exposed to FST one week after ovariectomy (63). In contrast with these studies, data from our laboratory indicated that subacute (3 days) or chronic (26 days) E_2_ treatment failed to induce antidepressant effects in middle-aged rats exposed to FST three weeks after ovariectomy; however, in these studies, treatment with the synthetic estrogen prolame effectively reduced depressive behavior (59, 65), indicating that the antidepressant effects of estrogens may depend on the type of estrogen.

##### 2.2.2.3 Antidepressant-like effect of estrogen restitution depends on timing of administration

It has been suggested that, the antidepressant-like effects of estrogens observed in the ovariectomized aged female rat model may depend on the time of treatment initiation. Indeed, there is evidence to suggest that ethinyl-estradiol induces an antidepressant effect in ovariectomized middle-aged rats (15 months) exposed to the FST when administered at 1 week after ovariectomy, but not after 3 weeks, suggesting that the administration of ethinyl-estradiol is a crucial factor that contributes to its antidepressant-like effect (63). The relevance of the time of hormone treatment after ovariectomy in middle-aged rats has also been studied by other groups; one study compared the effect of long-term versus short-term post-ovariectomy E_2_ replacement and found that, in middle-aged rats (20 months), one month of treatment immediately after ovariectomy was more effective at reducing depressive behavior in the FST than E_2_ treatment five months after surgery, suggesting that a delay in hormone treatment reduces their favorable effects (66). The importance of the delay in receiving an estrogen treatment was recently studied in our laboratory, where experiments were conducted in middle-aged ovariectomized rats; in this study, the antidepressant effect of E_2_ or prolame (sub-acute treatment) was evaluated 3, 8, 16 or 24 weeks after ovariectomy, with results showing that E_2_ did not induce an effect at any post ovariectomy time, in contrast, prolame had antidepressant effect only 3 weeks after ovariectomy (63). In agreement with this finding, another study found that ovariectomized middle-aged rats (12 months old) that received an E_2_ capsule immediately after ovariectomy did not show any reduction in depressive behavior when evaluated in the FST after two weeks of treatment (67).

##### 2.2.2.4 E_2_ synergizes with antidepressant drugs to alleviate depressive-like behaviors

Some researchers have hypothesized that there is a synergistic relationship between E_2_ and antidepressant drugs in the alleviation of depression-like behavior. Indeed, one study reported that ethinyl-estradiol synergized with citalopram (at non-effective doses) to induce antidepressant effects in ovariectomized middle-aged rats (15 months) one week after ovariectomy, as measured in the FST (63). Another study showed that the combination of ineffective doses of fluoxetine (1.25 mg/kg) and E_2_ (2.5 ug/rat) reduced depressive behavior in aged rats (12-14 m.o.) exposed to the FST, furthermore, the chronic treatment with the same fluoxetine and E_2_ doses reversed the anhedonic state in middle-aged rats exposed to the chronic mild stress paradigm (68). In contrast, a single injection of a low dose of estradiol valerate (1 mg/kg) failed to potentiate or shorten the latency of action of low doses of chronic citalopram (5 mg/kg) (64). These data suggest that estrogen presentation and/or the type of SSRI may influence the synergistic relationship between estrogens and SSRI to induce antidepressant effects in middle-aged female rats.

### 2.3 Ovariectomy as a model of surgical menopause

Natural menopause is a clinical term that indicates the end of the reproductive period in women; however, a similar physiological state can be induced by bilateral oophorectomy, which is commonly referred to as surgical menopause. Both types of menopause in women can cause low plasma and brain concentrations of E_2_, progesterone, and other steroid hormones, as well as a marked elevation of FSH concentrations. These changes can affect specific brain neurotransmitter systems and peripheral physiological processes, negatively affecting the quality of life (42). The long-term absence of steroid hormones is associated with physiological changes that predispose individuals to mood disorders such as anxiety and depression, among others (42). In natural menopause, such changes occur gradually, and require a long time to stabilize. However, in surgical menopause, they are established over a shorter period, thus influencing symptom severity (42, 69, 70).

In experimental animals, ovariectomy is commonly used to evaluate the effects of gonadal hormonal deprivation, mainly estrogens, and subsequent exogenous hormone treatments on the brain and periphery. Rodent anatomy differs from that of humans; for example, rodents have a bifurcated uterus called the uterine horn (48), and the ovaries are located at the posterolateral poles of the kidneys, each attached by a mesovarium to the dorsal body wall of the abdominal cavity (71). The selection of the method for ovariectomy is essential for the success of the surgery, particularly when the number of animals is small, and the duration of the experiment is short. In anesthetized rats, the ovaries can be removed by three methods, as described below:

1. A single midline dorsal skin incision, 3 cm long, is made between the middle of the back and the base of the tail. This allows access to the peritoneal cavity, in which the ovary can be observed surrounded by fat. Blood vessels must be ligated to prevent bleeding, after which the connection between the Fallopian tube and the uterine horn is cut, the ovary is removed, and the incision is sutured;.
2. A single ventral transverse incision is made on the middle part of the abdomen, further a small transverse peritoneal incision of 0.4 to 0.6 cm is made with surgical scalpel blade on the middle part of the abdomen slightly to the right, immediately adjacent to the second right nipple of the rat. After accessing the peritoneal cavity, the adipose tissue can be pulled until the right uterine tube and the ovary surrounded by a variable amount of fat can be identified and exteriorized by gentle retraction. This procedure is then repeated for the left ovary through the same incision. The wound is then closed in two layers (muscle and skin) using sterile sutures, the peritoneum and muscle with an absorbable suture, and the skin with a non-absorbable suture;.
3. A double dorsolateral incision is made approximately 1 cm long above the ovaries, with the use of dissecting scissors. The skin is cut, allowing access to the dorsal muscles and peritoneal cavity. Subsequently, the operation is performed in the same manner as modality 1. The muscle incisions do not require suturing, and skin wounds can be closed bilaterally with a single catgut suture (72, 73). Full recovery from ovariectomy can be achieved within one week. The reported advantages of transverse incisions in abdominal surgery include less pain and a low incidence of hernia formation (72–74).

Experimental interventions occur either at the time of ovariectomy or commence once E_2_ has reached a low to undetectable level in the plasma, which typically occurs within 1-2 weeks. Thus, estrogen can be administered after ovariectomy, alone or in combination with other ovarian hormones, with variations in the type of estrogen administered, dose, route of administration, and treatment duration, while controlling for interactions with endogenous steroid hormones since the ovaries have been removed (48).

#### 2.3.1 Ovariectomy induces depressive-like behaviors in young adult rats

Preclinical studies have shown that ovariectomy in the medium- and long-term reduces the plasma and brain concentrations of steroid hormones and other molecules, which can negatively impact brain function (75, 76). Similar to endocrine changes in women, FSH levels increase with time following ovariectomy in mice, and are higher at 4 weeks than at 1-2 weeks (77). In contrast, progesterone and E_2_ levels are lower at 4 weeks than at 1-2 weeks post-ovariectomy and reach undetectable levels at 3 and 15 months post-ovariectomy, respectively (77, 78). Notably, these hormonal changes have been associated with an increase in anxiety- and depression-like behaviors, which depend on the time elapsed after ovariectomy, a phenomenon termed the ‘post-ovariectomy timeframe’. Anxiety-like behavior was higher in rats at 12 weeks than at 3 weeks post-ovariectomy (79); interestingly, no significant changes on anxiety-like behavior with respect to rats in proestrus-estrus and metestrus-diestrus phases were observed 1-week post-ovariectomy; however, 3 weeks post-ovariectomy, high anxiety-like behavior was detected in comparison with that of rats in the proestrus-estrus phase. Similar levels of anxiety-like behavior were detected in rats at 6, 9, 12, and 15 weeks post-ovariectomy (80), which coincided with a reduction in E_2_ and progesterone levels. Moreover, in the FST, an increase in total time of immobility was detected 2 weeks post-ovariectomy (81), which is considered to indicate a depressive-like state; however, this behavior was higher at 6 weeks post-ovariectomy in rats in the proestrus-estrus and metestrus-diestrus phases, and was maintained at high but similar levels in rats at 9, 12, and 15 weeks post-ovariectomy (14, 80). Anxiety- and depression-like behaviors are associated with a reduction in the number of Fos-immunoreactive cells in the lateral septal nucleus, a brain structure involved in the physiopathology of affective disorders, as well as the therapeutic effects of antidepressant drugs (82). It is important to mention that changes in Fos immunoreactivity in lateral septum cells and anxiety- and depression-like behaviors associated with long-term ovariectomy in rats were reversed by E_2_ treatment (80), highlighting the role of these limbic structures (and possibly their connections) in the regulation of stress-linked affective-like behaviors in individuals with depression associated with sex hormones.

#### 2.3.2 E_2_ produces antidepressive-like effect in ovariectomized young rats

As ovariectomy induces drastic changes in neuronal, metabolic, and cardiovascular functions that emulate those shown by women subjected to bilateral oophorectomy in our laboratory and many others, we have used the ovariectomy model in young adult female rodents to understand the effects of ERT on depression-like behaviors and their underlying mechanisms (65, 83, 84). In investigations of the antidepressant-like effects of hormonal agents, researchers have used different tests to reveal behavioral and neurochemical disturbances related to clinical depression. In this regard, FST mice and rats eventually develop passive behavior (immobility) that reflects despair. Hormones and drugs with antidepressant-like activity show reduced immobility and increased active behavior (14, 85, 86). Ovariectomy increases depressive behavior in young adult rats; however, E_2_ replacement at physiological levels can reduce this behavior (87). Furthermore, studies using FST have confirmed that the antidepressant effects of estrogen depend on the type of estrogen, age, endocrine condition, and the initiation time of estrogen replacement after the estrogenic decline (88), termed the post-ovariectomy time frame. Clinical and preclinical studies have determined that the variability in the response to ERT may be partly due to a critical period or window of opportunity during which ERT must be started to alleviate depressed mood (89, 90).

#### 2.3.3 Antidepressant effect of estrogen depends on timing of administration in young adult

Estrada-Camarena et al. (91) found that the immobility behavior observed in young adult ovariectomized rats was reduced by acute E_2_ treatment when applied one or three weeks after surgery, but not after 12 weeks. In contrast, an acute treatment with the synthetic compound 17α-ethynyl-estradiol induced antidepressant-like actions even 12 weeks after ovariectomy, suggesting that the physicochemical features and bioavailability of each estrogen may modify their effectiveness as antidepressant drugs in a particular time frame of hormone deprivation (92). According to this notion, results obtained from our laboratory using a synthetic estrogen program have indicated its potential to reduce despair usefulness timeframes in which E_2_ is not further effective, particularly in middle-aged rats (59, 65). Together, these findings show that the antidepressant-like effects of estrogens in the model of surgical menopause are modulated by variables such as age and post-ovariectomy time frame, and the therapeutic potential of estrogens such as ethinyl-estradiol and prolame has been revealed in studies on the time frame of ovariectomy.

In this regard, the window of opportunity is related to the effects of estrogen on the structure and function of brain regions that regulate mood. One study carried out on rats ovariectomized with long hormone deprivation frames at 9- and 15-months showed increased dendritic spine density in the CA1 in response to E_2_, whereas longer hormone deprivation frames (19 months) did not (93). The loss of E_2_ efficacy in the 19-month post-ovariectomy group could be attributed to the duration of E_2_ deprivation rather than age, as old rats ovariectomized at 20 months of age responded to E_2_ replacement one month after ovariectomy when the rats were 21 months old (93). While structural changes in the CA1 region can be generalized to mood regulation, the behavioral results in the FST with E_2_ are contradictory (65, 66). This situation opens the opportunity to explore different aspects related to the HRT treatment of depression in menopause, such as the interplay between the post-ovariectomy time frame and different estrogen treatments, the metabolism of synthetic estrogens with potential as HRTs, and the role of additional brain areas or circuits involved in menopausal depression.

Recent studies using an ovariectomized rat model have been conducted to evaluate other substances with promising effects, thus expanding the therapeutic possibilities for alleviating depression in women (**Table 2**). In these studies, ovariectomized rodents treated with antidepressant drugs such as maprotiline, hormones such as progesterone and allopregnanolone, phytoestrogens like puerarin and genistein, dietary isoflavones, and flavonoids such as chrysin showed reduced depressive-like behavior (95–99) in the FST. Interestingly, at 4-weeks post-ovariectomy, rats treated with curcumin (100 mg/kg/4 weeks), a secondary metabolite extracted from *Curcuma longa*, showed reductions in the production of inflammatory cytokines and reestablished concentrations of dopamine, serotonin, and noradrenaline in brain structures related to depression (100). These beneficial effects were similar to those produced by E_2_ (100 μg/kg) and the antidepressant drug fluoxetine (20 mg/kg). Similar effects have been reported in 8–12 weeks post-ovariectomy rats treated with the flavonoid chrysin (1 mg/kg), which reduced the total time of immobility in the FST, similar to 1 mg/kg progesterone and 1 mg/kg allopregnanolone (96). These data show the utility of long-term ovariectomy on understanding the neurobiological basis underlying the long-term absence of ovarian hormones produced by ovariectomy and the identification of potential therapeutic substances for the treatment of depression symptoms associated with surgical menopause (14).

**Table 2:** Effects of timing post-ovariectomy and some pharmacological treatments on depression-like behaviors in mice and rats.

Animal/Weeks post-OVX	Effect of OVX on depression-like behavior / Test	Evaluated substance	Dosage	Treatment effect	Reference
Effect of several weeks post ovariectomy on depression-like behavior					
C57BL/6 mice / 2	No effect / CUS+FST	–	–	–	Lagunas et al., 2010
C57BL/6 mice / 2	No effect / FST Increases / TST	- -	- -	- -	Carrier et al., 2015
Wistar rats / 1, 3	No effect / FST	–	–	–	Puga-Olguín et al., 2019 (80)
Wistar rats / 8	No effect / FST	–	–	–	Dornellas et al., 2018
Wistar rats / 12, 60	No effect / FST	–	–	–	de Chaves et al., 2009 (78)
Wistar rats / 6, 9, 12, 15	Increases / FST	–	–	–	Puga-Olguín et al., 2019 (80)
C57BL/6 mice / 16	Increases / CUMS+FST	–	–	–	Lagunas et al., 2010
Effect of several hormones and other substances on depression-like behavior					
C57BL/6 mice /1-2	No effect / TST	Progesterone	10 mg/kg	Reduces depression-like behavior	Frye, 2011
Wistar rats / 2	Increases / FST	17β-estradiol Phytoestrogen genistein	0.5 μg/rat/2 weeks 10 mg/kg/2 weeks	Reduces depression- like behavior	Sapronov and Kasakova, 2008
Wistar rats / 3	Increases / FST	17β-estradiol Ethinyl-estradiol	5 and 10 μg/rat 2.5 and 5 μg/rat	Reduces depression- like behavior	Estrada-Camarena et al., 2003 (94)
Wistar rats / 3	Increases / FST	Ethinyl-estradiol	5 μg/rat	Reduces depression- like behavior	Vega-Rivera et al., 2013
Wistar rats / 2	No effect / FST	Progesterone	0.8, 1.6, and 3.0 mg/kg	Reduces depression-like behavior	Martínez-Mota et al., 1999
Wistar rats / 2	Increases / FST	17β-estradiol maprotiline	0.3, 1 and 3 μg/rat 0.6 mg/rat	Reduces depression-like behavior	Okada et al., 1997 (95)
Wistar rats / 2	Increases / FST	Progesterone, allopregnanolone, and chrysin	1 mg/kg	Reduces depression-like behavior	Cueto-Escobedo et al., 2020 (96)
Wistar rats / 7	Increases / FST	17β-estradiol	150 μg/rat/week	Reduces depression-like behavior	Diz-Chaves et al., 2012
Wistar rats / 8	Increases / FST	17β-estradiol	0.09 mg/kg	Reduces depression-like behavior	Puga-Olguín et al., 2019 (80)
Wistar rats / 8	Increases / FST	Progesterone	0.5, 1 and 2 mg/kg	Only 1 and 2 mg/kg P reduce depression-like behavior	Rodríguez-Landa et al., 2020 (97)
ICR mice / 8	Increases / FST Increases / TST	Phytoestrogen puerarin	1 μg/kg E2 and 100 mg/kg	Both treatments reduce depression-like behavior	Tantipongpiradet et al., 2019 (98)
Wistar rats / 8	Increases / FST	Progesterone	5 mg/kg	Reduces depression-like behavior	Rodríguez-Landa et al., 2020 (97)
Wistar rats / 9	Increases / FST	17β-estradiol	25 μg/day/6 weeks	Reduces depression-like behavior	Khayum et al., 2020 (99)
Wistar rats / 12	No effect / FST	17β-estradiol	0.25 mg/rat	No effect	Boldarine et al., 2019
Sprague-Dawley / 4	Increases / FST	17β-estradiol + dietary isoflavones	0.25 mg/kg/2 weeks	Reduces depression-like behavior	Russell et al., 2017

Behavioral test used to evaluate the effect of ovariectomy and drugs: CUS, chronic unpredictable stress; FST, forced swim test; TST, tail suspension test. OVX, ovariectomy.

Although ovariectomy appears to be the easiest model for conducting studies in rodents, this evidence demonstrates that this condition courses with dynamic changes at the molecular, cellular, and structural levels that modify the behavior and response to different stimuli. Knowledge of these processes is critical for the selection of a specific post-ovariectomy timeframe according to the study objectives. Evidence suggests that such a process may reveal or veil the effects of treatments, such as restitution with estrogens. In addition, the ovariectomy model has some limitations because the majority of women during the transition to menopause preserve their reproductive system intact, which must be considered when carrying out translational research. Second, the most common model of ovariectomy is in young adult animals, which usually exhibit regular estrous cycles before extirpation of the ovaries, suggesting important differences in the hormonal profile and regulation of the HPG axis compared to aged gonadally intact females. Finally, the age at which ovariectomy is performed and the period of hormonal deprivation (post-ovariectomy time) in rodents must be considered, as these factors may influence the sensitivity to hormonal treatment.

### 2.4 Accelerated ovarian failure as a model of menopause

Although they show great utility, the aged rat and ovariectomy models were unable to replicate all of the features of the transition to menopause experienced by middle-aged women. For example, the aged rat model does not allow the dissociation of the alterations related to the transition from those due to aging, whereas the ovariectomy model prevents females from gradually reducing ovarian function, as it favors the abrupt depletion of estrogens. This situation has generated an urgency to develop more adequate models that emulate the neuroendocrine changes and health risks linked to perimenopause. Certain classes of substances have been shown to produce early ovarian failure in women (101). One such compound is 4-vinyl cyclohexene diepoxide (VCD), a metabolite of 4-vinyl cyclohexene (VHC), used in the industrial production of diepoxides and epoxy resins. VCD is an organic volatile compound with alkylating properties that appears to be more potent than its parent compound in producing ovotoxic effects, suggesting that this metabolite is the active form of VHC (101). Two long-term studies have identified that both toxicants induced ovarian and uterine atrophy, accompanied by the loss of ovarian follicles and corpora lutea in female mice, indicating that infertility may occur as a result of the rapid exhaustion of primordial follicles. Notably, these studies identified that rats are sensitive to VCD, but not to VHC, which suggests that differences between species are probably associated with their metabolism (102).

It has previously been shown that VCD favors atresia of the primordial and primary follicles through the activation of apoptotic pathways involving the Bcl-2 family and caspase-3, as well as the mitogen activated protein kinase family (103, 104). Furthermore, studies using culture of ovaries of rats at 4PND, which are rich in primordial and primary follicles, identified that the KITLG neurotrophic factor reduced the ovotoxicity of VCD, which binds to its oocyte-associated receptor KIT, stimulating downstream signaling, whose function is oocyte growth and follicle survival. *In vitro* assays have further demonstrated that VCD directly inhibits autophosphorylation of the KIT receptor located on the plasma membrane of the oocyte, interfering with this signaling and producing atresia (105, 106).

#### 2.4.1 Ovarian failure induced by VCD in female rodents

The most common treatment for ovarian failure in mice and rats is the intraperitoneal administration of VCD at doses ranging from to 80-160 mg/kg, over at least 15 days (107, 108). Depending on the dose and duration of VCD treatment, lengthening of the estrous cycle may be observed, accompanied by fluctuating levels of E_2_, which, after a certain time, tend to decrease, while the levels of LH/FSH increase (109, 110). Interestingly, ovarian failure is elicited before an increase in gonadotropins can detected, confirming that the primary target of VCD is the ovaries (104, 108). A VCD-induced menopause model in rats has been recently explored, with longitudinal studies identifying an endocrine pattern similar to that experienced in women in perimenopausal transition. To summarize, at 100 days after treatment, VCD produced low levels of progesterone and anti-Mullerian hormone, reduced levels of androgens, no changes or increased levels of E_2_, and no changes in FSH and corticosterone levels (111). Changes in gonadal and pituitary hormones related to follicle depletion may account for the abnormalities in the behavior of rats evaluated using different tests of depressive-like behavior.

Studies in juvenile (usually postnatal day [PND] 28) female mice and rats have demonstrated that repeated doses of VCD administered over a longer period are necessary to induce the complete loss of reproductive capacity; females showed a significant reduction in primordial and primary follicles after treatment for 10 days, while all primordial and primary follicles were lost after a treatment of 20 days (107). In turn, doses administered over long periods also significantly reduced the onset of ovarian failure from 135 days post-treatment to 52 days post-treatment (107). This generates a significant reduction of time in the establishment of a menopause-like stage, relative to natural decline of ovarian function in middle-aged females. In addition, the identification of different stages of menopause (pre-, peri-, and post-menopause) in the model of VCD-induced ovarian failure would allow the characterization of neurobehavioral disturbances and their associated mechanisms at specific points in time, which is a frequent limitation of studies enrolling women. A possible restriction in the interpretation of the results is that natural estropause occurs at older ages, mainly in rats; hence, the earlier onset of ovarian failure induced by VCD appears to be indicative of the induction of premature or precocious menopause. Interestingly, recent reports have described that VCD can also induce ovarian failure in adult and middle-aged females (48, 109, 112), which strengthens the validity of VCD administration in modeling menopause.

#### 2.4.2 VCD-induced ovarian failure is related to depressive-like behaviors in rodents

In support of the notion of the existence of perimenopausal stage in the VCD model, mice treated with VCD developed abnormalities in the locomotion in the open field test, showing reductions in the time spent in the center and the distance traveled in the open field, which is indicative of high levels of anxiety. These females also showed high levels of corticosterone after exposure to the test and relative to the controls without VCD (113), revealing a facilitation in the HPA axis in peri-estropausal females in challenging conditions. Another study was designed to evaluate the time course of neurobehavioral disturbances in mice at 20, 35, and 52 days after treatment with VCD. A time-response effect was detected in the expression of depression-like behavior in the tail suspension test; females had higher levels of immobility for 35 days post VCD treatment, an effect that was maintained at 52 days post VCD treatment. In turn, high anxiety levels in the plus maze test were observed at 20 days post-VCD treatment relative to the control, gradually reducing to lower levels at 52 days post-treatment. Alterations in sleep were also observed in VCD-treated females, showing a reduction in REM sleep during the inactive and active periods. In these females, a remarkable reduction in the percentage of time spent in proestrus manifested mainly at 52 days post-VCD treatment, together with a decrease in ovarian and uterine indices (110). Similar to mice, a transversal study in rats treated with VCD showed that females exhibited a lower number of entries into the open arms of the elevated plus maze relative to controls, and increased time in the corner of an open field test, expressed in a consistent manner, and increased anxiety-like behavior in different tests (112, 114). Altogether, these findings strengthen the idea that VCD model rodents display set of signs that emulate menopausal mood symptoms.

Healthy cognitive systems are required to face daily life events. Hence, alterations in cognition may represent a challenge for women transitioning to menopause, increasing the risk of developing affective disorders. One study designed to induce follicular failure in middle-aged rats showed that females experienced an impairment of working memory that started in the early transition, which was amplified in mid- and post-follicular depletion. This observation attributed to reproductive perturbations rather than aging, as untreated age-matched controls did not exhibit cognitive failures (49). Longitudinal evaluation of these females revealed that in the post-follicular condition, middle-aged rats retained the impairment of working memory relative to younger VCD-treated animals, suggesting additive effects between age and ovarian failure on hippocampal-dependent memories. Interestingly, cognitive flexibility is not affected in younger or older females (49), indicating that some cognitive domains are more affected by the interruption of ovarian function than others. The results also implied that the different stages of transition to a non-reproductive stage had an impact on the severity of neurobehavioral disturbances.

With due caution, these abovementioned signs observed in VCD-treated females may support the notion of a causal relationship between the cessation of ovarian function and the cognitive, neurobehavioral, and mood symptoms experienced by women undergoing menopause.

#### 2.4.3 Effect of E_2_ in the depressive-like behaviors induced by VCD

Studies in rats have shown that HRT can reduce depression-like signs in females with ovarian failure. For example, middle-aged VCD rats (11-12 months-old) were treated for 21 days with E_2_ plus levonorgestrel, with results showing that this treatment regimen improved the entry of females to the center of an open field, indicating a reduction in anxiety, while combinations of E_2_ plus levonorgestrel and E_2_ plus progesterone were effective in reducing immobility behavior in the FST, denoting antidepressant-like effects (114). In addition, the cognitive evaluation of middle-aged VCD rats showed divergent results related to the E_2_ treatment schedule. Tonic administration of E_2_ via an osmotic pump over 12 days improved learning in 11-to 12-month-old rats (115); however, daily E_2_ injections were ineffective at improving learning in the water maze (114). The combination of E_2_ and levonorgestrel, but not E_2_ plus progesterone, was found to improve learning relative to vehicle, E_2_, progesterone, or levonorgestrel alone (114). These results indicate the important additive effects of estradiol and levonorgestrel in the treatment of depressive-like signs and cognitive symptoms in menopause. Levonorgestrel, a synthetic progestin, is a 19-nortestosterone derivative used in emergency contraception. Similar to progesterone, levonorgestrel exerts a significant androgenic activity (116), which may account for the behavioral differences detected in middle-aged females with ovarian failure. Altogether, these findings suggest that some hormone combinations used in menopausal hormone restitution may be more successful in managing affective symptoms in women with follicle-depleted ovaries than in those with surgical menopause.

#### 2.4.4 Effect of E_2_ in brain targets in the model of VCD

E_2_ interacts with the serotonergic system through several pathways to modulate depression (117). In the VCD model, ovarian failure was found to be related to the reduced expression of ER-β mRNA and tryptophan hydroxylase (TPH) immunoreactivity in the dorsal raphe nucleus (DRN), as well as low levels of serotonin in dorsal hippocampus, which were increased with a treatment of E_2_ (21 days, pellet implant) (118). One study further analyzed the changes in the CA1 hippocampal region in VCD-treated mice, which showed a reduced number of spines and terminals expressing ER-α with respect to the controls treated with the vehicle (119). ER-α is critical in the rapid regulation of NMDA receptors to facilitate neuroplasticity and neuroadaptation to environmental changes. Indeed, one study demonstrated that VCD reduced the serum levels of E_2_ and progesterone in follicle-depleted female mice, as well as serotonin synthesis in the DRN and the number of fibers arriving at the basolateral amygdala (120). These changes were accompanied by the facilitation of long-term potentiation mediated by glutamate release in the amygdala, which was restored to normal levels following treatment with E_2_ (120). It would be interesting to investigate the effect of a combination of E_2_ with progesterone and levonorgestrel, or even the effect of new estrogens proposed to regulate depression in menopause, serotonin levels, and ER in brain structures related to depression and mood regulation. Finally, questions regarding whether different estrogens in combination with antidepressant drugs are effective in reducing depression in females with accelerated ovarian failure remain unanswered.

## 3 Discussion

Preclinical studies have shown that the antidepressant-like effects of different estrogen treatments may vary depending on the animal model of menopause used in the investigation. The most consistent findings on the therapeutic potential of estrogens in depression have been found in the ovariectomized rat model, with E_2_, estradiol valerate, ethinyl estradiol, and prolame (63, 68, 84, 94). These effects were found independent of female age, as rats ranging from 2.5 to 15 months of age showed a response to estrogens including a reduction in despair or anhedonia. Preclinical studies have further suggested that factors such as the post-ovariectomy timeframe and timing of treatment may improve or limit the effectiveness of estrogen treatments in the middle-aged population (14, 63, 65–67, 80).

Notably, the model of the natural transition to estropause in middle-aged females is challenging in the sense that it produces greater variability in the antidepressant-like effect of estrogens, and may even cancel the actions of these treatments (59). Middle-aged intact females have several characteristic endocrine features, such as high and low serum levels of E_2_, low-to-moderate but persistent levels of progesterone, and low levels of androgens (121); estrogens and progesterone act coordinately through the ER and progesterone receptors, respectively, to regulate brain homeostasis in young cycling females. However, these cyclic changes in progesterone levels are lost in middle-aged females (121), suggesting that sustained levels of this steroid may alter the effects of estrogen on brain function. Varying levels of E_2_ can affect the distribution of intracellular and membrane estrogen receptors in the hippocampus of female rats (122), and these changes may interfere with the ability of estrogen treatment to alleviate depression. Interestingly, residual ovaries in gonadally-intact aged and VCD-treated females retain the synthesis of androgens (i.e., androstenedione), which are not present in ovariectomized females (109, 115), indicating the presence of a hormonal profile similar to that observed in postmenopausal women (54, 55). Such an androgenic state may render phenotypes with different sensitivities to respond with antidepressant-like behavior to ERT or estrogen in combination with progestins.

It has previously been reported that aging in females impairs the amplitude of day–night differences in multiunitary activity in brain regions that play a role in controlling biological rhythms (123), which affects the release of neurotransmitters that interact with estrogens to regulate mood and exert antidepressant effects. Studies using the accelerated ovarian failure model have confirmed that female mice transitioning to a post-follicular depletion phase exhibit diminished brain connectivity between the hippocampus and frontal cortex, in addition to a shorter latency to mount the wheel in the running wheel test, and an increased total activity in the wheel during the light periods when control animals sleep (124). Running in a wheel is an elective behavior that denotes motivation in rodents; hence, these results suggest that periestropausal mice are more driven to run to obtain rewards that alter normal wake-sleep cycles. These findings, together with alterations in serotonergic neurotransmission in the basolateral amygdala (120), the expression of different mRNA brain-derived neurotrophic factor exons, and the reduction of dendritic spines in hippocampal regions (119), support the notion that the progressive change from a reproductive to a non-reproductive stage in females may account for deficiencies in brain communication that affect affective-like behavior.

The results of our study in addition to others in the literature have demonstrated that ovariectomy in middle-aged rats cancels irregularities in endocrine oscillations, permitting the observation of the potential therapeutic effects of treatments such as estrogens and antidepressants. This model thus offers opportunities to evaluate the pharmacological and behavioral effects of treatments on time frames in the lives of females, in which classic hormonal and antidepressant treatments are not effective. In turn, the behavioral and physiological outcomes in the ovarian failure model confirmed its face validity and usefulness in research on the specific mechanisms associated with the transition to menopause.

### 3.1 Mechanism of depression in menopause models

The post-ovariectomy time frame induces neuroadaptations in different brain structures that participate in the regulation of depressive-like behavior and stress responses. One to three weeks after ovariectomy, a reduction in dendritic spines and synaptophysin density occurs in the pyramidal neurons of the CA1 layer of the hippocampus in rats, indicating structural changes in areas involved in the regulation of cognition and emotions (125, 126). At four weeks post-ovariectomy, neuroadaptations in GABA_A_ receptors can be inferred by a reduction in mRNA of the α2- and α3- subunits of this receptor in the amygdala, a region involved in expression of fear and emotional responses to stress. Interestingly, the 5 alpha-reduced metabolite of progesterone, allopregnanolone, is a ligand for sites in the GABA_A_ receptor that modulates depressive-like behavior in the FST (127, 128). It is possible that neuroadaptations in GABA_A_ play a role in the reduction of stress resilience in long-term ovariectomized females, as well as in the decline of interactions between estrogens and receptors associated with a poorer antidepressant response to estrogens. In agreement with changes in GABA_A_ receptors, serotonin neurotransmission in the superior areas appears to be interrupted at the post-ovariectomy timeframe of four weeks (129), since the mRNA of TPH and the serotonin transporter (SERT) is reduced in the DRN at this time (130). This change may be associated with reduced activation of structures, such as the lateral septum, which participates in the regulation of fear and response to antidepressant drugs (80). Interestingly, estrogen receptor subtypes decline as the time after post-ovariectomy increases. Studies have demonstrated a reduction in the mRNA levels of estrogen receptor subtypes in the hippocampus and prefrontal cortex of ovariectomized females, 8 to 9 weeks post-ovariectomy (131), in conjunction with a reduction in the antidepressant-like effect of different ERT, and the limited effects of some ERTs in middle-aged to old ovariectomized rats.

Many neurotransmitters, including monoamines, GABA, and glutamate, have all been associated with depressive-like behaviors and responses to ERTs in mice and rats. Interestingly, a new mechanism has emerged to explain the behavioral changes related to ovariectomy and hormone deprivation. In this regard, the mechanisms underlying central inflammation seem to play a major role in depression-like behaviors. Ovariectomy in adult female C57BL/6 mice was found to increase depression- and anxiety-like behaviors five weeks after surgery in the FST (132), sucrose preference test, tail suspension test, open field test, and elevated zero maze. Those behavioral changes occurred simultaneously to an increase in pro-IL-1β and pro-IL-18; IL-1b and IL-18 protein and mRNA in hippocampus but not in the prefrontal cortex and amygdala (133). NLRP3 inflammasome components increased in OVX mice, including NLRP3 mRNA, and cleaved caspase-1 P10 (active caspase-1) protein, but not the ASC protein; followed by increased in the level of TLR-2 and TLR-4, active NF-kB which regulates the production of cytokines. These results have been replicated in Sprague-Dawley rats 10 weeks after ovariectomy, with results showing an induction in anxiety- and depression-like behaviors in the FST, sucrose preference test, elevated plus maze, and novelty-suppressed feeding test, while simultaneously increasing proinflammatory cytokines, neural apoptosis and microglial activation from immunoregulatory to proinflammatory phenotype in the hippocampus and reinforced NFκB (134, 135).

Ovariectomy-induced depression- and anxiety-like behaviors, as well as increases in inflammatory proteins can be reversed by E_2_ and ER-β agonist, but not ER-α agonist treatments in mice (133). Additionally, experiments in female Sprague–Dawley rats with a post-ovariectomy timeframe of 8 weeks showed that ovariectomy triggered high levels of depression-like behaviors in the tail suspension test associated with a decrease in BDNF levels in the hippocampus but not in the prefrontal cortex; these alterations were ameliorated by 8 weeks of exercise on a treadmill or E_2_ administration (136). Changes in cytokine levels in the prefrontal cortex have only been observed in Wistar rats 9-11-weeks post-ovariectomy, resulting in the induction of heart failure. These rats further had higher levels of IL-2 and IL-6 and decreased mature BDNF in the prefrontal cortex compared to intact rats and ovariectomized rats without heart failure; E_2_ replacement or the pro-inflammatory cytokine synthesis inhibitor pentoxifylline ameliorated these changes at the experimental level (137, 138).

In mice, ovariectomy triggered cognitive impairments in variables of the Y-Maze, Novel object recognition and Morris water maze test at 30-60 days post-surgery. These results were associated with increases of malondialdehyde (a product of lipid oxidation), a reduction of superoxide dismutase and catalase activity in hippocampus and a significant increase in mRNA of proinflammatory cytokines (IL-1β, IL-6, and tumor necrosis factor α), whereas mRNA expression of the estrogen-mediated gene PI3K decreased in the hippocampus of OVX mice compared with sham controls (139). Similar results were observed in the Y maze test in 4-month-old Sprague-Dawley rats 12 weeks after ovariectomy, with impairments linked to the TLR4/NF-κB inflammatory pathway and excessive activation of microglia in the hippocampus (140). Both cognitive impairment and hippocampal alterations have been observed in preclinical and clinical studies of depression.

### 3.2 Antidepressant mechanisms of estrogens – evidence from menopause animal models

In women, the effects of E_2_ have been observed in brain regions known to be closely involved in mood regulation (141). Evidence suggests that the modulatory mood effects of estrogen are largely dependent on changes in the serotonergic pathways. As E_2_ can regulate gene expression after coupling with estrogen receptors (142), which are expressed within the midbrain raphe nucleus (the main reservoir of serotonergic neurons) (143, 144), this treatment may modulate the expression of genes that regulate serotonin neurotransmission implicated in depression. E_2_ has been implicated in increased serotonin synthesis, decreased serotonin breakdown, and modulation of serotonergic receptors (145). Thus, assessment the close relationship between E_2_ and the serotonergic system may provide insight into why some women experience increased susceptibility to mood symptoms during periods of hormonal fluctuation.

There is significant evidence from rodent models of menopause to suggest that the antidepressant properties of estrogens are mediated by the intracellular content of ER-α or ER-β (84), which are distributed in areas relevant to affective disorders (limbic cortices, amygdala, hippocampus, and hypothalamus) that are innervated by serotonergic and noradrenergic neurons (146). The mechanism by which ERs exert these effects is transcriptional; when activated, these receptors bind to estrogen response elements in the promoter region of genes relevant for antidepressant effects (117, 147–149).

Several hypotheses have been proposed to explain the development depressive disorders and their treatment, including monoaminergic, chemical, and neural network hypotheses, which are complementary and may explain the different steps of antidepressant drug action (150). Remarkably, the evidence suggests that E_2_ modulates the mechanisms involved in each hypothesis.

#### 3.2.1 Estrogens modulate monoaminergic neurotransmission

According to the monoaminergic hypothesis, depression is caused by a deficiency in central monoamines (serotonin, noradrenaline, and dopamine), which may be restored by antidepressants. The evidences indicates that estrogens increase the serotoninergic tone by several presynaptic mechanisms: a) increasing the expression of TPH, the rate limiting enzymes in the synthesis of serotonin, in raphe nuclei (151–153); b) down-regulating the activity of monoamine oxidase (MAO), an enzyme that breaks down serotonin, in the hypothalamus and amygdala of female ovariectomized rats (154); c) reducing serotonin autoinhibition through the down-regulation of the serotonin-1A receptor (5HT_1A_) mRNA and serotonin-1B receptor (5HT_1B_) mRNA autoreceptors in the hippocampus (dentate gyrus and CA2 region) and dorsal raphe nuclei (155, 156).

In addition to presynaptic effects, E_2_ has been shown to modulate postsynaptic 5HT_1A_ and 5HT_2A_ receptors, which are the most studied receptors in relation to affective disorders. In this regard, the female Flinders Sensitive Line, a genetic animal model of depression, showed lower serotonin-2A receptor (5-HT_2A_) mRNA expression in the perirhinal cortex, piriform cortex, and medial amygdala than in control rats (157). Ovariectomy further decreased the levels of 5HT_2A_ mRNA receptor in the prefrontal cortex of female Fisher rats (158), and E_2_ treatment increased 5HT_2A_ mRNA and receptor binding in the cortico-limbic areas of ovariectomized female rats (158, 159). Regarding 5HT_1A_ receptors, E_2_ treatment downregulates mRNA levels in the cingulate, piriform, and perirhinal cortices, and the amygdala (157, 160), and decreased 5HT_1A_ receptor binding in the hippocampus, amygdala, and prefrontal cortex (161, 162). These data indicate that E_2_ up-regulates postsynaptic 5HT_2A_ receptors and down-regulates postsynaptic 5HT_1A_ receptors, thereby contributing to central serotonergic neurotransmission. In addition, E_2_ increases SERT expression in the DRN of female rat brains (163).

ER-β expression has been detected in noradrenergic neurons in the locus coeruleus (164), as well as dopaminergic neurons in the ventral tegmental area and substantia nigra (165). As such, E_2_ can influence noradrenergic and dopaminergic neurotransmission. In support of this, this hormone stimulates the gene expression of the enzymes required for noradrenaline synthesis (tyrosine hydroxylase and dopamine β-hydroxylase) in the rat locus coeruleus (166), and increases the activity of striatal tyrosine hydroxylase (167). Additionally, estrogen increases noradrenaline turnover, enhances its release in the hypothalamus (168), increases the release of dopamine from the central amygdaloid nucleus (169) and increases dopamine D2 receptor expression in the striatum, nucleus accumbens, and dopamine transporter in the middle striatum (170, 171). Finally, experiments using human cell culture have demonstrated that E_2_ decreases the protein levels of catechol-O methyltransferase, an enzyme that degrades noradrenaline and dopamine (172).

Overall, these data indicate that E_2_ can increase monoamine levels by enhancing the capacity for synthesis while reducing degradation.

#### 3.2.2 Estrogens stimulate BDNF synthesis

The chemical hypothesis of depression suggests that the antidepressant effect of relevant drugs is related to the increased expression of neurotrophic factors such as BDNF; in this context, estrogens have been shown to upregulate BDNF in the rat hippocampus (173–175). Although a putative estrogen response element has been identified within the BDNF gene (174), the colocalization of ER and BDNF is sparse (α) or null (β) in the hypothalamus, amygdala, prelimbic cortex, and ventral hippocampus of female rats (176). It has further been reported that cortical ER-β is almost exclusively localized to parvalbumin-immunoreactive GABAergic neurons (177), while further analysis through double labeling of ER-β/parvalbumin indicated that axons from cortical ER-β inhibitory neurons innervated BDNF immunoreactive pyramidal neurons, suggesting a transsynaptic regulation of BDNF synthesis (176). Furthermore, a more recent study from the same group identified that estrogens may regulate BDNF expression by a two-step transsynaptic mechanism as follows: estrogen up-regulates GABA levels in ER-β expressing cells, which inhibits the non-bearing ER GABAergic neurons, leading to the downregulation of GABA. This effect causes the disinhibition of BDNF-producing neurons and increases BDNF expression (178).

The neurotrophic factor IGF-I is another molecule involved in the antidepressant response (179) whose its activity is regulated by estrogens. In support of this, studies have shown that ovariectomy down-regulates IGF-I receptor densities in the hippocampus, hypothalamus, and parietal cortex, and E_2_ treatment restores IGF-I receptor levels (180).

#### 3.2.3 Estrogens induce neurogenesis and synaptic plasticity

The neural network depression hypothesis posits that antidepressant treatments restore neuronal connectivity through modulating neuroplasticity. The colocalization of Ki-67 mRNA (an endogenous cell proliferation marker) and either ER-β mRNA or ER-α mRNA in the dentate gyrus suggests a direct influence of ERs on cell proliferation in female rats (180). In this regard, the administration of E_2_ or ER−α and ER−β selective agonists (propyl pyrazole triol, PPT, and diarylpropionitrile, DPN, respectively) induces cell proliferation in the adult rat dentate gyrus (181–183). In addition, ovariectomy decreases cell proliferation and expression of PSA-NCAM (a neural cell adhesion molecule expressed by granule cells during differentiation) in the dentate gyrus of female rats, while a single injection of E_2_ completely reverses these effects (184). The effect of estrogen on hippocampal neurogenesis may be partially explained by its effects on monoaminergic neurotransmission and neurotrophic factors, as suggested by evidence indicating that the inhibition of serotonin synthesis prevents the effect of E_2_ on hippocampal cell proliferation (184), and that BDNF and IGF-I, neurotrophic factors regulated by estrogens, may increase the survival and proliferation of newborn cells in the dentate gyrus (185–187).

In addition to neurogenesis, estrogen induces synaptic plasticity. Activation of ER-β increases key synaptic proteins, such as PSD-95, synaptophysin, and the AMPA receptor subunit GluR1, in the hippocampus of mice. In addition, ER-β activation enhances LTP in hippocampal slices and increases dendritic branching and spine density in hippocampal neurons, and improved cognitive performance has been observed in several memory tests (188).

The effects of E_2_ on the serotonergic system have been reproduced in a primate model of menopause. In this study, long-term loss of E_2_ in ovariectomized monkeys was found to decrease the global availability of this monoamine, and was associated with fewer serotonergic neurons and a reduced expression of TPH-2, SERT, and 5-HT_1A_ receptors compared to intact animals (189). In addition, E_2_ treatment in ovariectomized macaques increased the expression of TPH-2, MAO-A, and MAO-B mRNA (190). Furthermore, the estrogen postsynaptic effects found in rat models agree with the findings in menopausal women; by downregulating 5-HT_1A_ auto-receptors and upregulating 5-HT_2A_ receptors, E_2_ increases serotonin availability for postsynaptic transmission (6). Thus, these data demonstrate the translational validity of the rat menopause models.

FST has been used to elucidate the primary mechanisms of action of antidepressants, with research showing that antidepressants acting on the serotonergic system increase swimming behavior, whereas those interacting with the noradrenergic or dopaminergic systems facilitate climbing behavior (85). The data obtained in our laboratory indicate that estrogen (E_2_ and prolame) treatment significantly increased swimming behavior in young rats at 3 and 8 weeks after ovariectomy without modifying climbing behavior. The same effect was observed in middle-aged rats three weeks after ovariectomy treated only with prolame (65), suggesting that the antidepressant-like effects of both estrogens are related to the regulation of the serotonergic system, in agreement with the mechanisms described above, possibly by activating ER. Our data further support the idea that under long-term estrogen deprivation or advanced age, the inefficacy of some estrogenic treatments (such as E_2_ and prolame) in inducing antidepressant-like effects is related, at least partially, to their inability to improve serotoninergic system activity.

### 3.3 Stress and estrogens in menopausal depression

Exposure to psychosocial factors (8, 19), either in early life or currently (191), is a strong predictor of depression in midlife. Once the stress axis is activated, the neurons of the paraventricular nucleus (PVN) are stimulated to release peptides, such as corticotropin-releasing hormone (CRH) and vasopressin (AVP), which act on the corticotropes of the anterior pituitary to stimulate the release of the adrenocorticotropic hormone (ACTH) into the portal system. ACTH activates its receptors in the adrenal glands, eliciting the release of cortisol (in humans) and corticosterone (in rodents) to cope with environmental demands. High levels of corticosterone bind to receptors in the PVN, thus decreasing the release of peptides through negative feedback, and controlling HPA activity. Interactions between stress and the estrogen system may be related to the dysregulation of brain function in females with erratic estrogen levels, including as in perimenopause. Corticosterone reduces the release of CRH from the PVN, while E_2_ increases, whereas chronic exposure to high levels of corticosterone interferes with estrogen release, indicating a bidirectional relationship between the HPA and HPG axes. In rats, ER-β is the predominant form in the PVN, and the ER subtype is most closely related to the anxiolytic- and antidepressant-like effects of estrogens (192). Treatment with estrogens reduces ER-β activity in the nucleus, whereas treatment with synthetic glucocorticoids increases the expression of this receptor (193). The physiological relevance and translation of these results to the vulnerability to stress in perimenopause is complex; however, one interpretation is that higher levels of glucocorticoids would increase the sensitivity of the PVN neurons to estrogens, facilitating their actions, while high levels of estrogens would reduce the sensitivity of these neurons, controlling the effects of estrogens on the HPA axis (193).

Serotonergic neurons in the DRN, which co-express glucocorticoid receptors (GR) and ER-β, have been implicated in the stress response (194). GR and ER-β have opposing effects on depressive behavior. In ovariectomized animal models subjected to the FST, administration physiological dosages of E_2_ and DPN activated ER-β, decreased depressive behavior, increased the mRNA expression of TPH in the DRN, and promoted swimming behavior (195). In turn, the GR responds to high levels of corticosterone and induces depressive behavior in rats, while glucocorticoid treatment decreases TPH mRNA levels in the raphe nuclei (196). Interestingly, high dosage E_2_ can increase corticosterone levels by about two to three-fold, in addition to enhancing the action of corticosterone mainly through ER-α but not through ER-β (195). In addition, studies using various dosages of E_2_ have reported that SERT mRNA is upregulated in the DRN of ovariectomized rats (163). These studies have indicated that ovariectomy or hypoestrogenic stages are related to serotonin deficiency, which facilitates maladaptive stress responses and the development of depression.

Altogether, there is strong evidence to suggest that the interaction discussed in this section may play a role in the higher sensitivity to estrogen fluctuations experienced by perimenopausal women and would account for an increase in anxiety and greater risk of depression. In support of this idea, association studies in women showed that the longer exposure to estrogens fluctuations were positively associated with increased risk of perimenopausal depression (197, 198). The effects of estrogenic treatments, alone or in combination with antidepressants, should be evaluated based on these hypotheses.

### 3.4 Considerations for the research of depression in menopause in animal models

Animal models are useful in the investigation of menopause under highly controlled conditions, allowing the exploration of the causal effects of isolated variables; however, menopause has many complex multifactorial influences. For example, a higher prevalence of vasomotor symptoms (more frequent and problematic hot flashes) during menopause has been associated with higher temperatures and lower altitudes, but not with seasonal variations in temperature, in Spanish-speaking women from Santiago de Chile (Chile), Guayaquil and Quito (Ecuador), Panama City (Panamá), and Madrid (Spain) (199). In another example, Korean women aged 45–69 years with healthy dietary habits (high intake of whole-grain rice, legumes, vegetables, fruits, and fish) were found to have a significantly lower risk of depressive symptoms, as measured with the Beck Depression Inventory-II (200), while exercise decreased alterations in bone mineral density, sleep, anxiety, depression, and fatigue in peri- and post-menopausal women (201). Psychological factors such as personality, self-esteem, and coping skills, as well as relationship issues and social support, may contribute to the onset, course, and repose to perimenopausal period (202). All these factors pose a challenge in preclinical research on menopause and highlight the need for and importance of personalized medicine in clinical populations.

## 4 Conclusions

Overall, the significant evidence discussed in this review demonstrates the validity and utility of different models of menopause in the investigation of depression and its treatment. Findings from the ovariectomy model highlight the importance of this model in the detection of new hormone therapies with potential antidepressant-like activity. In turn, models of early ovarian failure and naturally aged females have advantages in the study of the transition to a non-reproductive stage characterized by depleted but hormonally active (i.e., releasing androgens) ovarian follicles. The selection of a specific model may depend on the organismic and pharmacological variables relevant to the investigation of menopausal depression.

Endocrine changes related to menopause produce an imbalance in steroid hormones, neurotransmitter systems, oxidative stress, and neuroinflammatory processes, together with changes in neuroanatomical structures, such as the raphe nucleus, hippocampus, prefrontal cortex, hypothalamus, amygdala, and lateral septal nucleus, involved in stress, anxiety, and depression (42, 203, 204). These modifications follow a time course that must be considered for the proposed investigation and constitute a scientific basis for explaining the critical window during which estrogen replacement is beneficial for alleviating depression and its symptoms. In this regard, this review also shows that the effectiveness of estrogen treatments in reducing depression in menopause is modulated by factors such as the stage of menopause (i.e., transitioning to menopause or post-menopause), timing of treatment initiation, and type and duration of treatment with estrogens.

A comparison of findings from different models of menopause revealed the complexity of dynamic changes occurring in brain functions during the transition, reinforcing the idea that the best approach is timely intervention considering the opportunity window, with careful selection of treatment according to the presence (or absence) of reproductive tissue. In this regard, there is evidence to suggest that several new promising estrogens could be considered ERTs with antidepressant properties highlighting their actions in endocrine conditions in which traditional ERTs are not effective. Overall, menopause is a condition for which personalized medicine is strongly needed, while the investigation of classic and new molecules in different models of menopause is required to expand the possibilities of treatment to increase resilience to stress and alleviate depressive symptoms in women.

## Author contributions

JH-P: Conceptualization, Data curation, Investigation, Writing – original draft. OH-H: Investigation, Writing – original draft, Methodology. MF-R: Investigation, Writing – original draft, Methodology. JC-E: Data curation, Investigation, Writing – original draft. JR-L: Conceptualization, Investigation, Writing – original draft, Writing – review & editing. LM-M: Conceptualization, Data curation, Formal analysis, Funding acquisition, Investigation, Project administration, Supervision, Writing – original draft, Writing – review & editing.

## References

[1] American Psychiatric Association. Diagnostic and Statistical Manual of Mental Disorders. 5th ed. Washington DC, USA: American Psychiatric Publishing (2013). doi: 10.1176/appi.books.9780890425596

[2] Williams ES Mazei-Robison M Robison AJ. Sex differences in major depressive disorder (MDD) and preclinical animal models for the study of depression. Cold Spring Harb Perspect Biol. (2022). doi: 10.1101/cshperspect.a039198 PMC8886985 34404738

[3] Di Benedetto MG Landi P Mencacci C Cattaneo A. Depression in women: Potential biological and sociocultural factors driving the sex effect. Neuropsychobiology. (2024) 83:2–16. doi: 10.1159/000531588 38272005 PMC10871691

[4] Joffe H de Wit A Coborn J Crawford S Freeman M Wiley A. Impact of estradiol variability and progesterone on mood in perimenopausal women with depressive symptoms. J Clin Endocrinol Metab. (2020) 105:e642–50. doi: 10.1210/clinem/dgz181 PMC7075107 31693131

[5] Joffe H Crawford SL Freeman MP White DP Bianchi MT Kim S. Independent contributions of nocturnal hot flashes and sleep disturbance to depression in estrogen-deprived women. J Clin Endocrinol Metab. (2016) 101:3847–55. doi: 10.1210/jc.2016-2348 PMC5052351 27680875

[6] Soares CN. Depression in peri- and postmenopausal women: Prevalence, pathophysiology and pharmacological management. Drugs Aging. (2013) 30:677–85. doi: 10.1007/s40266-013-0100-1 23801148

[7] Uddenberg ER Safwan N Saadedine M Hurtado MD Faubion SS Shufelt CL. Menopause transition and cardiovascular disease risk. Maturitas. (2024) 185:107974. doi: 10.1016/j.maturitas.2024.107974 38555760

[8] Bromberger JT Kravitz HM. Mood and menopause: findings from the Study of Women's Health Across the Nation (SWAN) over 10 years. Obstet Gynecol Clin North Am. (2011) 38:609–25. doi: 10.1016/j.ogc.2011.05.011 PMC3197240 21961723

[9] Zakaria R Al Rahbi B Ahmad AH Said RM Othman Z Azman KF. Menopause rodent models: Suitability for cognitive aging research. Intern Med J. (2019) 26:450–2.

[10] Harlow SD Gass M Hall JE Lobo R Maki P Rebar RW. Executive summary of the Stages of Reproductive Aging Workshop + 10: Addressing the unfinished agenda of staging reproductive aging. J Clin Endocrinol Metab. (2012) 97:1159–68. doi: 10.1210/jc.2011-3362 PMC3319184 22344196

[11] Bellino FL Wise PM. Nonhuman primate models of menopause workshop. Biol Reprod. (2003) 68:10–8. doi: 10.1095/biolreprod.102.005215 12493689

[12] Medina-Contreras J Villalobos-Molina R Zarain-Herzberg A Balderas-Villalobos J. Ovariectomized rodents as a menopausal metabolic syndrome model. A minireview Mol Cell Biochem. (2020) 475:261–76. doi: 10.1007/s11010-020-03879-4 32852713

[13] Bimonte-Nelson HA Bernaud VE. How preclinical models of menopause can inform clinical care: A focus on midlife and reciprocal communication between clinical and preclinical science. Curr Opin Endocr Metab Res. (2023) 28:100434. doi: 10.1016/j.coemr.2023.100434 PMC11526845 39484630

[14] Rodríguez-Landa JF. Considerations of timing post-ovariectomy in mice and rats in studying anxiety- and depression-like behaviors associated with surgical menopause in women. Front Behav Neurosci. (2022) 16:829274. doi: 10.3389/fnbeh.2022.829274 35309685 PMC8931748

[15] Gallez A Nys G Wuidar V Dias Da Silva I Taziaux M Kinet V. Comparison of estetrol exposure between women and mice to model preclinical experiments and anticipate human treatment. Int J Mol Sci. (2023) 24:9718. doi: 10.3390/ijms24119718 37298669 PMC10253893

[16] Hong KH Jung J Kim M Um MY. Hyperoside ameliorates depression-like behavior in ovariectomized mice. Appl Biol Chem. (2024) 67:41. doi: 10.1186/s13765-024-00897-4

[17] Wattanathorn J Thukham-Mee W. Omega-3-rich tuna oil derived from by-products of the canned tuna industry enhances memory in an ovariectomized rat model of menopause. Antioxidants. (2024) 13:637. doi: 10.3390/antiox13060637 38929077 PMC11201088

[18] Jia Y Zhou Z Xiang F Hu W Cao X. Global prevalence of depression in menopausal women: A systematic review and meta-analysis. J Affect Disord. (2024) 358:474–82. doi: 10.1016/j.jad.2024.05.051 38735578

[19] Freeman EW Sammel MD Liu L Gracia CR Nelson DB Hollander L. Hormones and menopausal status as predictors of depression in women in transition to menopause. Arch Gen Psychiat. (2004) 61:62–70. doi: 10.1001/archpsyc.61.1.62 14706945

[20] Bromberger JT Matthews KA Schott LL Brockwell S Avis NE Kravitz HM. Depressive symptoms during the menopausal transition: The Study of Women's Health Across the Nation (SWAN). J Affect Disord. (2007) 103:267–72. doi: 10.1016/j.jad.2007.01.034 PMC2048765 17331589

[21] Schmidt PJ Haq N Rubinow DR. A longitudinal evaluation of the relationship between reproductive status and mood in perimenopausal women. Am J Psychiat. (2004) 161:2238–44. doi: 10.1176/appi.ajp.161.12.2238 15569895

[22] Harnod T Chen W Wang JH Lin SZ Ding DC. Hysterectomies are associated with an increased risk of depression: A population-based cohort study. J Clin Med. (2018) 7:366. doi: 10.3390/jcm7100366 30340333 PMC6210125

[23] Hickey M Schoenaker DA Joffe H Mishra GD. Depressive symptoms across the menopause transition: Findings from a large population-based cohort study. Menopause. (2016) 23:1287–93. doi: 10.1097/GME.0000000000000712 27552471

[24] Rocca WA Grossardt BR Geda YE Gostout BS Bower JH Maraganore DM. Long-term risk of depressive and anxiety symptoms after early bilateral oophorectomy. Menopause. (2008) 15:1050–9. doi: 10.1097/gme.0b013e318174f155 18724263

[25] Rocca WA Grossardt BR Geda YE Gostout BS Bower JH Maraganore DM. Long-term risk of depressive and anxiety symptoms after early bilateral oophorectomy. Menopause. (2018) 25:1275–85. doi: 10.1097/GME.0000000000001229 30358723

[26] Bräuner EV Wilson LF Koch T Christensen J Dehlendorff C Duun-Henriksen AK. The long-term association between bilateral oophorectomy and depression: A prospective cohort study. Menopause. (2022) 29:276–83. doi: 10.1097/GME.0000000000001913 35213515

[27] Lin KY Chou CY Chang CY Lin WC Wan L. Association between oophorectomy and depression in patients with comorbidities: A nationwide cohort study in Taiwan. Taiwan J Obstet Gynecol. (2020) 59:899–905. doi: 10.1016/j.tjog.2020.09.017 33218409

[28] Joffe H Massler A Sharkey KM. Evaluation and management of sleep disturbance during the menopause transition. Semin Reprod Med. (2010) 28:404–21. doi: 10.1055/s-0030-1262900 PMC3736837 20845239

[29] Kulkarni J Gurvich C Mu E Molloy G Lovell S Mansberg G. Menopause depression: Under recognized and poorly treated. Aust N Z J Psychiat. (2024), 48674241253944. doi: 10.1177/00048674241253944 PMC11308326 38761367

[30] Seib C Anderson D Lee K. Prevalence and correlates of sleep disturbance in postmenopausal women: the Australian Healthy Aging of Women (HOW) Study. J Womens Health (Larchmt). (2014) 23:151–8. doi: 10.1089/jwh.2013.4472 24261649

[31] Jeon GH. Insomnia in postmenopausal women: How to approach and treat it? J Clin Med. (2024) 13:428. doi: 10.3390/jcm13020428 38256562 PMC10816958

[32] Bromberger JT Epperson CN. Depression during and after the perimenopause: Impact of hormones, genetics, and environmental determinants of disease. Obstet Gynecol Clin North Am. (2018) 45:663–78. doi: 10.1016/j.ogc.2018.07.007 PMC6226029 30401549

[33] Mosconi L Berti V Dyke J Schelbaum E Jett S Loughlin L. Menopause impacts human brain structure, connectivity, energy metabolism, and amyloid-beta deposition. Sci Rep. (2021) 11:10867. doi: 10.1038/s41598-021-90084-y 34108509 PMC8190071

[34] Ramli NZ Yahaya MF Mohd Fahami NA Abdul Manan H Singh M Damanhuri HA. Brain volumetric changes in menopausal women and its association with cognitive function: A structured review. Front Aging Neurosci. (2023) 15:1158001. doi: 10.3389/fnagi.2023.1158001 37818479 PMC10561270

[35] Ryan J Burger HG Szoeke C Lehert P Ancelin ML Henderson VW. A prospective study of the association between endogenous hormones and depressive symptoms in postmenopausal women. Menopause. (2009) 16:509–17. doi: 10.1097/gme.0b013e31818d635f PMC2814239 19169164

[36] Wnuk A Przepiórska K Pietrzak BA Kajta M. Emerging evidence on membrane estrogen receptors as novel therapeutic targets for central nervous system pathologies. Int J Mol Sci. (2023) 24:4043. doi: 10.3390/ijms24044043 36835454 PMC9968034

[37] García-Segura LM Chowen JA Párducz A Naftolin F. Gonadal hormones as promoters of structural synaptic plasticity: Cellular mechanisms. Prog Neurobiol. (1994) 44:279–307. doi: 10.1016/0301-0082(94)90042-6 7886228

[38] Spencer-Segal JL Tsuda MC Mattei L Waters EM Romeo RD Milner TA. Estradiol acts via estrogen receptors alpha and beta on pathways important for synaptic plasticity in the mouse hippocampal formation. Neuroscience. (2012) 202:131–46. doi: 10.1016/j.neuroscience.2011.11.035 PMC3505616 22133892

[39] González-Burgos I Velázquez-Zamora DA González-Tapia D. Estradiol-mediated modulation of memory and of the underlying dendritic spine plasticity through the life span. Histol Histopathol. (2024) 39:411–23. doi: 10.14670/HH-18-672 37966087

[40] Inagaki T Etgen AM. Neuroprotective action of acute estrogens: animal models of brain ischemia and clinical implications. Steroids. (2013) 78:597–606. doi: 10.1016/j.steroids.2012.12.015 23385013 PMC3733348

[41] García-Ríos RI Mora-Pérez A Soria-Fregozo C. Depression and serotonergic changes during the climacteric and postmenopausal stages: Hormonal influences. In: Rodriguez-Landa JF Cueto-Escobedo J, editors. A Multidisciplinary Look at Menopause. Croatia: InTech (2017). p. 63–80. doi: 10.5772/intechopen.69786

[42] Giannini A Caretto M Genazzani AR Simoncini T. Neuroendocrine changes during menopausal transition. Endocrines. (2021) 2:405–16. doi: 10.3390/endocrines2040036

[43] Marsh WK Bromberger JT Crawford SL Leung K Kravitz HM Randolph JF. Lifelong estradiol exposure and risk of depressive symptoms during the transition to menopause and postmenopause. Menopause. (2017) 24:1351–9. doi: 10.1097/GME.0000000000000929 PMC5860642 28719421

[44] Schmidt PJ Ben Dor R Martinez PE Guerrieri GM Harsh VL Thompson K. Effects of estradiol withdrawal on mood in women with past perimenopausal depression: A randomized clinical trial. JAMA Psychiat. (2015) 72:714–26. doi: 10.1001/jamapsychiatry.2015.0111 PMC6391160 26018333

[45] Rubinow DR Johnson SL Schmidt PJ Girdler S Gaynes B. Efficacy of estradiol in perimenopausal depression: So much promise and so few answers. Depress Anxiety. (2015) 32:539–49. doi: 10.1002/da.2015.32.issue-8 PMC6309886 26130315

[46] Gordon JL Rubinow DR Eisenlohr-Moul TA Xia K Schmidt PJ Girdler SS. Efficacy of transdermal estradiol and micronized progesterone in the prevention of depressive symptoms in the menopause transition: A randomized clinical trial. JAMA Psychiatry. (2018) 75:149–57. doi: 10.1001/jamapsychiatry.2017.3998 PMC5838629 29322164

[47] Graziottin A Serafini A. Depression and the menopause: Why antidepressants are not enough? Menopause Int. (2009) 15:76–81. doi: 10.1258/mi.2009.009021 19465674

[48] Koebele SV Bimonte-Nelson HA. Modeling menopause: The utility of rodents in translational behavioral endocrinology research. Maturitas. (2016) 87:5–17. doi: 10.1016/j.maturitas.2016.01.015 27013283 PMC4829404

[49] Diaz Brinton R. Minireview: translational animal models of human menopause: challenges and emerging opportunities. Endocrinology. (2012) 153:3571–8. doi: 10.1210/en.2012-1340 PMC3404353 22778227

[50] Finch CE Felicio LS Mobbs CV Nelson JF. Ovarian and steroidal influences on neuroendocrine aging processes in female rodents. Endocrinol Rev. (1984) 5:467–97. doi: 10.1210/edrv-5-4-467 6389107

[51] Finch CE. The menopause and aging, a comparative perspective. J Steroid Biochem Mol Biol. (2014) 142:132–41. doi: 10.1016/j.jsbmb.2013.03.010 PMC3773529 23583565

[52] Clemens JA Meites J. Neuroendocrine status of old constant-estrous rats. Neuroendocrinology. (1971) 7:249–56. doi: 10.1159/000121973 4927853

[53] Huang HH Steger RW Bruni JF Meites J. Patterns of sex steroid and gonadotropin secretion in aging female rats. Endocrinology. (1978) 103:1855–9. doi: 10.1210/endo-103-5-1855 570913

[54] Fogle RH Stanczyk FZ Zhang X Paulson RJ. Ovarian androgen production in postmenopausal women. J Clin Endocrinol Metab. (2007) 92:3040–3. doi: 10.1210/jc.2007-0581 17519304

[55] Nunes E Gallardo E Morgado-Nunes S Fonseca-Moutinho J. Steroid hormone levels in postmenopausal hysterectomised women with and without ovarian conservation: the continuous endocrine function of the ovaries. J Obstet Gynaecol. (2023) 43:2141618. doi: 10.1080/01443615.2022.2141618 36331514

[56] Wang J Yu R Han QQ Huang HJ Wang YL Li HY. G-1 exhibit antidepressant effect, increase of hippocampal ERs expression and improve hippocampal redox status in aged female rats. Behav Brain Res. (2019) 359:845–52. doi: 10.1016/j.bbr.2018.07.017 30041006

[57] Mahmoud R Wainwright SR Chaiton JA Lieblich SE Galea LAM. Ovarian hormones, but not fluoxetine, impart resilience within a chronic unpredictable stress model in middle-aged female rats. Neuropharmacology. (2016) 107:278–93. doi: 10.1016/j.neuropharm.2016.01.033 27018449

[58] Brotto LA Gorzalka BB Barr AM. Paradoxical effects of chronic corticosterone on forced swim behaviours in aged male and female rats. Eur J Pharmacol. (2001) 424:203–9. doi: 10.1016/S0014-2999(01)01148-7 11672564

[59] Lemini C García-Albor E Cruz-López B Matamoros-Trejo G Márquez-Baltazar S Herrera-Pérez JJ. Prolame produces anxiolytic- and antidepressant-like effects in middle-aged female rats with less uterotrophic effects than 17β-estradiol. Eur J Pharmacol. (2024) 969:176454. doi: 10.1016/j.ejphar.2024.176454 38417607

[60] Fernández-Guasti A Olivares-Nazario M Reyes R Martínez-Mota L. Sex and age differences in the antidepressant-like effect of fluoxetine in the forced swim test. Pharmacol Biochem Behav. (2017) 152:81–9. doi: 10.1016/j.pbb.2016.01.011 26807812

[61] Rasmussen DD. Physiological interactions of the basic rest–activity cycle of the brain: Pulsatile luteinizing hormone secretion as a model. Psychoneuroendocrinology. (1986) 11:389–405. doi: 10.1016/0306-4530(86)90001-6 3550855

[62] Scarbrough K Wise PM. Age-related changes in pulsatile luteinizing hormone release precede the transition to estrous acyclicity and depend upon estrous cycle history. Endocrinology. (1990) 126:884–90. doi: 10.1210/endo-126-2-884 2404750

[63] Vega Rivera NM Gallardo Tenorio A Fernández-Guasti A Estrada Camarena E. The post-ovariectomy interval affects the antidepressant-like action of citalopram combined with ethynyl-estradiol in the forced swim test in middle aged rats. Pharm (Basel). (2016) 9:21. doi: 10.3390/ph9020021 PMC4932539 27153072

[64] Romano-Torres M Fernández-Guasti A. Estradiol valerate elicits antidepressant-like effects in middle-aged female rats under chronic mild stress. Behav Pharmacol. (2010) 21:104–11. doi: 10.1097/FBP.0b013e328337bdfc 20168212

[65] Hernández-Hernández OT Herrera-Pérez JJ Chavira R Lemini C Martínez-Mota L. Effects of post-ovariectomy time frame and age on the antidepressant-like actions of estradiol and prolame in female rats. Horm Behav. (2022) 141:105154. doi: 10.1016/j.yhbeh.2022.105154 35306314

[66] Walf AA Paris JJ Frye CA. Chronic estradiol replacement to aged female rats reduces anxiety-like and depression-like behavior and enhances cognitive performance. Psychoneuroendocrinology. (2009) 34:909–16. doi: 10.1016/j.psyneuen.2009.01.004 PMC2696690 19216030

[67] Kiss A Delattre AM Pereira SI Carolino RG Szawka RE Anselmo-Franci JA. 17β-estradiol replacement in young, adult and middle-aged female ovariectomized rats promotes improvement of spatial reference memory and an antidepressant effect and alters monoamines and BDNF levels in memory- and depression-related brain areas. Behav Brain Res. (2012) 227:100–8. doi: 10.1016/j.bbr.2011.10.047 22085882

[68] Récamier-Carballo S Estrada-Camarena E Reyes R Fernández-Guasti A. Synergistic effect of estradiol and fluoxetine in young adult and middle-aged female rats in two models of experimental depression. Behav Brain Res. (2012) 233:351–8. doi: 10.1016/j.bbr.2012.05.034 22652396

[69] Rodríguez-Landa JF Puga-Olguín A Germán-Ponciano LJ García-Ríos RI Soria-Fregozo C. Anxiety in natural and surgical menopause-physiologic and therapeutic bases. In: Rodriguez-Landa JF Cueto-Escobedo J, editors. A Multidisciplinary look at Menopause. Croatia: InTech (2017). p. 173–98. doi: 10.5772/60621

[70] Kingsberg SA Larkin LC Liu JH. Clinical effects of early or surgical menopause. Obstet Gynecol. (2020) 135:853–68. doi: 10.1097/AOG.0000000000003729 32168205

[71] Souza VR Mendes E Casaro M Antiorio ATFB Oliveira FA Ferreira CM. Description of ovariectomy protocol in mice. Methods Mol Biol. (2019) 1916:303–9. doi: 10.1007/978-1-4939-8994-2_29 30535707

[72] Lasota A Danowska-Klonowska D. Experimental osteoporosis–different methods of ovariectomy in female white rats. Rocz Akad Med Bialymst. (2004) 49 Suppl 1:129–31. 15638397

[73] Khajuria DK Razdan R Mahapatra DR. Description of a new method of ovariectomy in female rats. Rev Bras Reumatol. (2012) 52:462–70. 22641600

[74] Luengo-Mateos M González-Vila A Torres Caldas AM Alasaoufi AM González-Domínguez M López M. Protocol for ovariectomy and estradiol replacement in mice. STAR Protoc. (2024) 5:102910. doi: 10.1016/j.xpro.2024.102910 38416648 PMC10907206

[75] Alagwu EA Nneli RO. Effect of ovariectomy on the level of plasma sex hormones in albino rats. Niger J Physiol Sci. (2005) 20:90–4. 17220934

[76] Lu H Wu Q Liu Z Li S. Impact of ovariectomy on neurotransmitter receptors BDNF/TrkB and endoplasmic reticulum molecular chaperones in rat hypoglossal nucleus. Sleep Biol Rhythms. (2024) 22: 363–372. doi: 10.1007/s41105-024-00520-5 38962802 PMC11217223

[77] Moiety FM Salem HA Mehanna RA Abdel-Ghany BS. Comparative study on induction and effects of surgical menopause in a female rat model: A prospective case control study. Int J Clin Exp Med. (2015) 8:9403–11. PMC4538166 26309602

[78] de Chaves G Moretti M Castro AA Dagostin W da Silva GG Boeck CR. Effects of long-term ovariectomy on anxiety and behavioral despair in rats. Physiol Behav. (2009) 97:420–5. doi: 10.1016/j.physbeh.2009.03.016 19321148

[79] Picazo O Estrada-Camarena E Hernandez-Aragon A. Influence of the post-ovariectomy time frame on the experimental anxiety and the behavioural actions of some anxiolytic agents. Eur J Pharmacol. (2006) 530:88–94. doi: 10.1016/j.ejphar.2005.11.024 16356491

[80] Puga-Olguín A Rodríguez-Landa JF Rovirosa-Hernández MJ Germán-Ponciano LJ Caba M Meza E. Long-term ovariectomy increases anxiety- and despair-like behaviors associated with lower Fos immunoreactivity in the lateral septal nucleus in rats. Behav Brain Res. (2019) 360:185–95. doi: 10.1016/j.bbr.2018.12.017 30529405

[81] Fedotova J Dudnichenko T Kruzliak P Puchavskaya Z. Different effects of vitamin D hormone treatment on depression-like behavior in the adult ovariectomized female rats. BioMed Pharmacother. (2016) 84:1865–72. doi: 10.1016/j.biopha.2016.10.107 27847201

[82] Contreras CM Rodríguez-Landa JF Gutiérrez-García AG Bernal-Morales B. The lowest effective dose of fluoxetine in the forced swim test significantly affects the firing rate of lateral septal nucleus neurons in the rat. J Psychopharmacol. (2001) 15:231–6. doi: 10.1177/026988110101500401 11769815

[83] Estrada-Camarena E López-Rubalcava C Vega-Rivera N Récamier-Carballo S Fernández-Guasti A. Antidepressant effects of estrogens: A basic approximation. Behav Pharmacol. (2010) 21:451–64. doi: 10.1097/FBP.0b013e32833db7e9 20700047

[84] Lemini C Cruz-López B Martínez-Mota L. Participation of estrogen receptors in the antidepressant-like effect of prolame on the forced swimming test. Pharmacol Biochem Behav. (2013) 103:659–65. doi: 10.1016/j.pbb.2012.11.004 23159542

[85] Detke MJ Rickels M Lucki I. Active behaviors in the rat forced swimming test differentially produced by serotonergic and noradrenergic antidepressants. Psychopharmacol (Berl). (1995) 121:66–72. doi: 10.1007/BF02245592 8539342

[86] Porsolt RD Anton G Blavet N Jalfre M. Behavioural despair in rats: a new model sensitive to antidepressant treatments. Eur J Pharmacol. (1978) 47:379–91. doi: 10.1016/0014-2999(78)90118-8 204499

[87] Walf AA Frye CA. Antianxiety and antidepressive behavior produced by physiological estradiol regimen may be modulated by hypothalamic-pituitary-adrenal axis activity. Neuropsychopharmacology. (2005) 30:1288–301. doi: 10.1038/sj.npp.1300708 15756306

[88] Sherwin BB. The critical period hypothesis: Can it explain discrepancies in the oestrogen-cognition literature? J Neuroendocrinol. (2007) 19:77–81. doi: 10.1111/j.1365-2826.2006.01508.x 17214869

[89] Acosta JI Hiroi R Camp BW Talboom JS Bimonte-Nelson HA. An update on the cognitive impact of clinically-used hormone therapies in the female rat: Models, mazes, and mechanisms. Brain Res. (2013) 1514:18–39. doi: 10.1016/j.brainres.2013.01.016 23333453 PMC3739440

[90] Sherwin BB Henry JF. Brain aging modulates the neuroprotective effects of estrogen on selective aspects of cognition in women: A critical review. Front Neuroendocrinol. (2008) 29:88–113. doi: 10.1016/j.yfrne.2007.08.002 17980408

[91] Estrada-Camarena E Marquez-Baltazar S López-Rubalcava C. Influence of postovariectomy time on the antidepressant-like actions of 17 beta-estradiol and ethynil-estradiol in the Forced Swimming Test. In: Neuroscience Meeting Planner. Society for Neuroscience, Washington, DC (2008). p. 56.3/BB8.

[92] Estrada-Camarena E López-Rubalcava C Hernández-Aragón A Mejía-Mauries S Picazo O. Long-term ovariectomy modulates the antidepressant-like action of estrogens, but not of antidepressants. J Psychopharmacol. (2011) 25:1365–77. doi: 10.1177/0269881111408456 21890587

[93] Smith CC Vedder LC Nelson AR Bredemann TM McMahon LL. Duration of estrogen deprivation, not chronological age, prevents estrogen's ability to enhance hippocampal synaptic physiology. Proc Natl Acad Sci USA. (2010) 107:19543–8. doi: 10.1073/pnas.1009307107 PMC2984203 20974957

[94] Estrada-Camarena E Fernández-Guasti A López-Rubalcava C. Antidepressant-like effect of different estrogenic compounds in the forced swimming test. Neuropsychopharmacology. (2003) 28:830–8. doi: 10.1038/sj.npp.1300097 12637949

[95] Okada M Hayashi N Kometani M Nakao K Inukai T. Influences of ovariectomy and continuous replacement of 17beta-estradiol on the tail skin temperature and behavior in the forced swimming test in rats. Jpn J Pharmacol. (1997) 73:93–6. doi: 10.1254/jjp.73.93 9032138

[96] Cueto-Escobedo J Andrade-Soto J Lima-Maximino M Maximino C Hernández-López F Rodríguez-Landa JF. Involvement of GABAergic system in the antidepressant-like effects of chrysin (5,7-dihydroxyflavone) in ovariectomized rats in the forced swim test: Comparison with neurosteroids. Behav Brain Res. (2020) 386:112590. doi: 10.1016/j.bbr.2020.112590 32184157

[97] Rodríguez-Landa JF Olmos-Vázquez OJ Dutra da Costa BP Lima-Maximino M Maximino C Guillén-Ruiz G. Actions of progesterone on depression-like behavior in a model of surgical menopause are mediated by GABA_A_ receptors. Salud Mental. (2020) 43:43–53. doi: 10.17711/SM.0185-3325.2020.007

[98] Tantipongpiradet A Monthakantirat O Vipatpakpaiboon O Khampukdee C Umehara K Noguchi H. Effects of puerarin on the ovariectomy-induced depressive-like behavior in ICR mice and its possible mechanism of action. Molecules. (2019) 24:4569. doi: 10.3390/molecules24244569 31847138 PMC6943479

[99] Khayum MA Moraga-Amaro R Buwalda B Koole M den Boer JA Dierckx RAJO. Ovariectomy-induced depressive-like behavior and brain glucose metabolism changes in female rats are not affected by chronic mild stress. Psychoneuroendocrinology. (2020) 115:104610. doi: 10.1016/j.psyneuen.2020.104610 32088632

[100] Saied NM Georgy GS Hussien RM Hassan WA. Neuromodulatory effect of curcumin on catecholamine systems and inflammatory cytokines in ovariectomized female rats. Clin Exp Pharmacol Physiol. (2021) 48:337–46. doi: 10.1111/1440-1681.13427 33098686

[101] Oktem O Kim SS Selek U Schatmann G Urman B. Ovarian and uterine functions in female survivors of childhood cancers. Oncologist. (2018) 23:214–24. doi: 10.1634/theoncologist.2017-0201 PMC5813745 29158370

[102] Smith BJ Mattison DR Sipes IG. The role of epoxidation in 4-vinylcyclohexene-induced ovarian toxicity. Toxicol Appl Pharmacol. (1990) 105:372–81. doi: 10.1016/0041-008X(90)90141-G 2237912

[103] Springer LN McAsey ME Flaws JA Tilly JL Sipes IG Hoyer PB. Involvement of apoptosis in 4-vinylcyclohexene diepoxide-induced ovotoxicity in rats. Toxicol Appl Pharmacol. (1996) 139:394–401. doi: 10.1006/taap.1996.0180 8806857

[104] Springer LN Flaws JA Sipes IG Hoyer PB. Follicular mechanisms associated with 4-vinylcyclohexene diepoxide-induced ovotoxicity in rats. Reprod Toxicol. (1996) 10:137–43. doi: 10.1016/0890-6238(95)02056-X 8919611

[105] Kappeler CJ Hoyer PB. 4-Vinylcyclohexene diepoxide: a model chemical for ovotoxicity. Syst Biol Reprod Med. (2012) 58:57–62. doi: 10.3109/19396368.2011.648820 22239082 PMC3307534

[106] Mark-Kappeler CJ Sen N Lukefahr A McKee L Sipes IG Konhilas J. Inhibition of ovarian KIT phosphorylation by the ovotoxicant 4-vinylcyclohexene diepoxide in rats. Biol Reprod. (2011) 85:755–62. doi: 10.1095/biolreprod.111.092742 PMC3184290 21677306

[107] Lohff JC Christian PJ Marion SL Hoyer PB. Effect of duration of dosing on onset of ovarian failure in a chemical-induced mouse model of perimenopause. Menopause. (2006) 13:482–8. doi: 10.1097/01.gme.0000191883.59799.2e 16735946

[108] Van Kempen TA Milner TA Waters EM. Accelerated ovarian failure: A novel, chemically induced animal model of menopause. Brain Res. (2011) 1379:176–87. doi: 10.1016/j.brainres.2010.12.064 PMC3078694 21211517

[109] Brooks HL Pollow DP Hoyer PB. The VCD mouse model of menopause and perimenopause for the study of sex differences in cardiovascular disease and the metabolic syndrome. Physiol (Bethesda). (2016) 31:250–7. doi: 10.1152/physiol.00057.2014 PMC5504385 27252160

[110] Yu S Zhang L Wang Y Yan J Wang Q Bian H. Mood, hormone levels, metabolic and sleep across the menopausal transition in VCD-induced ICR mice. Physiol Behav. (2023) 265:114178. doi: 10.1016/j.physbeh.2023.114178 37001841

[111] Carolino ROG Barros PT Kalil B Anselmo-Franci J. Endocrine profile of the VCD-induced perimenopausal model rat. PloS One. (2019) 14:e0226874. doi: 10.1371/journal.pone.0226874 31887176 PMC6936812

[112] Reis FM Pestana-Oliveira N Leite CM Lima FB Brandão ML Graeff FG. Hormonal changes and increased anxiety-like behavior in a perimenopause-animal model induced by 4-vinylcyclohexene diepoxide (VCD) in female rats. Psychoneuroendocrinology. (2014) 49:130–40. doi: 10.1016/j.psyneuen.2014.06.019 25080405

[113] Kim D Liu QF Jeong HJ Han SH Kim DI Jeon S. A modified formulation of sutaehwan ameliorates menopausal anxiety, depression and heart hypertrophy in the VCD-induced menopausal mouse model. Biol Pharm Bull. (2019) 42:1471–81. doi: 10.1248/bpb.b19-00056 31474708

[114] Koebele SV Hiroi R Plumley ZMT Melikian R Prakapenka AV Patel S. Clinically used hormone formulations differentially impact memory, anxiety-like, and depressive-like behaviors in a rat model of transitional menopause. Front Behav Neurosci. (2021) 15:696838. doi: 10.3389/fnbeh.2021.696838 34366807 PMC8335488

[115] Koebele SV Mennenga SE Poisson ML Hewitt LT Patel S Mayer LP. Characterizing the effects of tonic 17β-estradiol administration on spatial learning and memory in the follicle-deplete middle-aged female rat. Horm Behav. (2020) 126:104854. doi: 10.1016/j.yhbeh.2020.104854 32949557 PMC8032560

[116] Schindler AE Campagnoli C Druckmann R Huber J Pasqualini JR Schweppe KW. Classification and pharmacology of progestins. Maturitas. (2008) 61:171–80. doi: 10.1016/j.maturitas.2008.11.013 19434889

[117] Hernández-Hernández OT Martínez-Mota L Herrera-Pérez JJ Jiménez-Rubio G. Role of estradiol in the expression of genes involved in serotonin neurotransmission: Implications for female depression. Curr Neuropharmacol. (2019) 17:459–71. doi: 10.2174/1570159X16666180628165107 PMC6520586 29956632

[118] Pestana-Oliveira N Kalil B Leite CM Carolino ROG Debarba LK Elias LLK. Effects of estrogen therapy on the serotonergic system in an animal model of perimenopause induced by 4-vinylcyclohexen diepoxide (VCD). eNeuro. (2018) 5:e0247–17.2017. doi: 10.1523/ENEURO.0247-17.2017 PMC5777542 29362726

[119] Van Kempen TA Gorecka J Gonzalez AD Soeda F Milner TA Waters EM. Characterization of neural estrogen signaling and neurotrophic changes in the accelerated ovarian failure mouse model of menopause. Endocrinology. (2014) 155:3610–23. doi: 10.1210/en.2014-1190 PMC4138565 24926825

[120] Wang Y Liu Y Xiong J Di T Yuan Z Wu J. Reduced serotonin impairs long-term depression in basolateral amygdala complex and causes anxiety-like behaviors in a mouse model of perimenopause. Exp Neurol. (2019) 321:113030. doi: 10.1016/j.expneurol.2019.113030 31377402

[121] Dudley EC Hopper JL Taffe J Guthrie JR Burger HG Dennerstein L. Using longitudinal data to define the perimenopause by menstrual cycle characteristics. Climacteric. (1998) 1:18–25. doi: 10.3109/13697139809080677 11907922

[122] Adams MM Fink SE Shah RA Janssen WG Hayashi S Milner TA. Estrogen and aging affect the subcellular distribution of estrogen receptor-alpha in the hippocampus of female rats. J Neurosci. (2002) 22:3608–14. doi: 10.1523/JNEUROSCI.22-09-03608.2002 PMC6758372 11978836

[123] Nakamura TJ Nakamura W Yamazaki S Kudo T Cutler T Colwell CS. Age-related decline in circadian output. J Neurosci. (2011) 31:10201–5. doi: 10.1523/JNEUROSCI.0451-11.2011 PMC3155746 21752996

[124] Vrontou S Bédécarrats A Wei X Ayodeji M Brassai A Molnár L. Altered brain rhythms and behaviour in the accelerated ovarian failure mouse model of human menopause. Brain Commun. (2022) 4:fcac166. doi: 10.1093/braincomms/fcac166 35794872 PMC9253886

[125] McLaughlin KJ Baran SE Wright RL Conrad CD. Chronic stress enhances spatial memory in ovariectomized female rats despite CA3 dendritic retraction: possible involvement of CA1 neurons. Neuroscience. (2005) 135:1045–54. doi: 10.1016/j.neuroscience.2005.06.083 PMC1380305 16165283

[126] Velázquez-Zamora DA González-Tapia D González-Ramírez MM Flores-Soto ME Vázquez-Valls E Cervantes M. Plastic changes in dendritic spines of hippocampal CA1 pyramidal neurons from ovariectomized rats after estradiol treatment. Brain Res. (2012) 1470:1–10. doi: 10.1016/j.brainres.2012.06.012 22750586

[127] Estrada-Camarena E Contreras CM Saavedra M Luna-Baltazar I López-Rubalcava C. Participation of the lateral septal nuclei (LSN) in the antidepressant-like actions of progesterone in the forced swimming test (FST). Behav Brain Res. (2002) 134:175–83. doi: 10.1016/S0166-4328(02)00023-2 12191804

[128] Rodríguez-Landa JF Contreras CM García-Ríos RI. Allopregnanolone microinjected into the lateral septum or dorsal hippocampus reduces immobility in the forced swim test: Participation of the GABAA receptor. Behav Pharmacol. (2009) 20:614–22. doi: 10.1097/FBP.0b013e328331b9f2 19752723

[129] Pandaranandaka J Poonyachoti S Kalandakanond-Thongsong S. Anxiolytic property of estrogen related to the changes of the monoamine levels in various brain regions of ovariectomized rats. Physiol Behav. (2006) 87:828–35. doi: 10.1016/j.physbeh.2006.02.002 16545402

[130] Charoenphandhu N Nuntapornsak A Wongdee K Krishnamra N Charoenphandhu J. Upregulated mRNA levels of SERT, NET, MAOB, and BDNF in various brain regions of ovariectomized rats exposed to chronic aversive stimuli. Mol Cell Biochem. (2013) 375:49–58. doi: 10.1007/s11010-012-1527-0 23208077

[131] Jin M Jin F Zhang L Chen Z Huang H. Two estrogen replacement therapies differentially regulate expression of estrogen receptors alpha and beta in the hippocampus and cortex of ovariectomized rat. Brain Res Mol Brain Res. (2005) 142:107–14. doi: 10.1016/j.molbrainres.2005.09.013 16290139

[132] Wang Y Xu Y Sheng H Ni X Lu J. Exercise amelioration of depression-like behavior in OVX mice is associated with suppression of NLRP3 inflammasome activation in hippocampus. Behav Brain Res. (2016) 307:18–24. doi: 10.1016/j.bbr.2016.03.044 27036651

[133] Xu Y Sheng H Bao Q Wang Y Lu J Ni X. NLRP3 inflammasome activation mediates estrogen deficiency-induced depression- and anxiety-like behavior and hippocampal inflammation in mice. Brain Behav Immun. (2016) 56:175–86. doi: 10.1016/j.bbi.2016.02.022 26928197

[134] Zhang WY Guo YJ Wang KY Chen LM Jiang P. Neuroprotective effects of vitamin D and 17ß-estradiol against ovariectomy-induced neuroinflammation and depressive-like state: Role of the AMPK/NF-κB pathway. Int Immunopharmacol. (2020) 86:106734. doi: 10.1016/j.intimp.2020.106734 32604067

[135] Wu B Song Q Zhang Y Wang C Yang M Zhang J. Antidepressant activity of ω-3 polyunsaturated fatty acids in ovariectomized rats: role of neuroinflammation and microglial polarization. Lipids Health Dis. (2020) 19:4. doi: 10.1186/s12944-020-1185-2 31915015 PMC6950787

[136] Lu J Xu Y Hu W Gao Y Ni X Sheng H. Exercise ameliorates depression-like behavior and increases hippocampal BDNF level in ovariectomized rats. Neurosci Lett. (2014) 573:13–8. doi: 10.1016/j.neulet.2014.04.053 24813109

[137] Najjar F Ahmad M Lagace D Leenen FHH. Sex differences in depression-like behavior and neuroinflammation in rats post-MI: role of estrogens. Am J Physiol Heart Circ Physiol. (2018) 315:H1159–73. doi: 10.1152/ajpheart.00615.2017 PMC6297826 30052050

[138] Najjar F Ahmad M Lagace D Leenen FHH. Role of myocardial infarction-induced neuroinflammation for depression-like behavior and heart failure in ovariectomized female rats. Neuroscience. (2019) 415:201–14. doi: 10.1016/j.neuroscience.2019.07.017 31351141

[139] Chulikhit Y Sukhano W Daodee S Putalun W Wongpradit R Khamphukdee C. Effects of *Pueraria candollei* var *mirifica* (Airy Shaw and Suvat.) Niyomdham on ovariectomy-induced cognitive impairment and oxidative stress in the mouse brain. Molecules. (2021) 26:3442. doi: 10.3390/molecules26113442 34198932 PMC8201258

[140] Song C Zhang Y Cheng L Shi M Li X Zhang L. Tea polyphenols ameliorates memory decline in aging model rats by inhibiting brain TLR4/NF-κB inflammatory signaling pathway caused by intestinal flora dysbiosis. Exp Gerontol. (2021) 153:111476. doi: 10.1016/j.exger.2021.111476 34265410

[141] Deecher D Andree TH Sloan D Schechter LE. From menarche to menopause: exploring the underlying biology of depression in women experiencing hormonal changes. Psychoneuroendocrinology. (2008) 33:3–17. doi: 10.1016/j.psyneuen.2007.10.006 18063486

[142] Paech K Webb P Kuiper GG Nilsson S Gustafsson J Kushner PJ. Differential ligand activation of estrogen receptors ERalpha and ERbeta at AP1 sites. Science. (1997) 277:1508–10. doi: 10.1126/science.277.5331.1508 9278514

[143] Alves SE Weiland NG Hayashi S McEwen BS. Immunocytochemical localization of nuclear estrogen receptors and progestin receptors within the rat dorsal raphe nucleus. J Comp Neurol. (1998) 391:322–34. doi: 10.1002/(ISSN)1096-9861 9492203

[144] Sheng Z Kawano J Yanai A Fujinaga R Tanaka M Watanabe Y. Expression of estrogen receptors (alpha, beta) and androgen receptor in serotonin neurons of the rat and mouse dorsal raphe nuclei; sex and species differences. Neurosci Res. (2004) 49:185–96. doi: 10.1016/j.neures.2004.02.011 15140561

[145] Lokuge S Frey BN Foster JA Soares CN Steiner M. Depression in women: windows of vulnerability and new insights into the link between estrogen and serotonin. J Clin Psychiatry. (2011) 72:e1563–9. doi: 10.4088/JCP.11com07089 22127200

[146] Nestler EJ Barrot M DiLeone RJ Eisch AJ Gold SJ Monteggia LM. Neurobiology of depression. Neuron. (2002) 34:13–25. doi: 10.1016/S0896-6273(02)00653-0 11931738

[147] Maharjan S Serova L Sabban EL. Transcriptional regulation of tyrosine hydroxylase by estrogen: opposite effects with estrogen receptors alpha and beta and interactions with cyclic AMP. J Neurochem. (2005) 93:1502–14. doi: 10.1111/j.1471-4159.2005.03142.x 15935066

[148] Bangasser DA Wiersielis KR Khantsis S. Sex differences in the locus coeruleus-norepinephrine system and its regulation by stress. Brain Res. (2016) 1641:177–88. doi: 10.1016/j.brainres.2015.11.021 PMC4875880 26607253

[149] Xie W Hong H Yang NN Lin RJ Simon CM Stallcup MR. Constitutive activation of transcription and binding of coactivator by estrogen-related receptors 1 and 2. Mol Endocrinol. (1999) 13:2151–62. doi: 10.1210/mend.13.12.0381 10598588

[150] Castrén E. Is mood chemistry? Nat Rev Neurosci. (2005) 6:241–6. doi: 10.1038/nrn1629 15738959

[151] Gundlah C Alves SE Clark JA Pai LY Schaeffer JM Rohrer SP. Estrogen receptor-beta regulates tryptophan hydroxylase-1 expression in the murine midbrain raphe. Biol Psychiatry. (2005) 57:938–42. doi: 10.1016/j.biopsych.2005.01.014 15820717

[152] Hiroi R McDevitt RA Neumaier JF. Estrogen selectively increases tryptophan hydroxylase-2 mRNA expression in distinct subregions of rat midbrain raphe nucleus: association between gene expression and anxiety behavior in the open field. Biol Psychiatry. (2006) 60:288–95. doi: 10.1016/j.biopsych.2005.10.019 16458260

[153] Donner N Handa RJ. Estrogen receptor beta regulates the expression of tryptophan-hydroxylase 2 mRNA within serotonergic neurons of the rat dorsal raphe nuclei. Neuroscience. (2009) 163:705–18. doi: 10.1016/j.neuroscience.2009.06.046 PMC2740745 19559077

[154] Holschneider DP Kumazawa T Chen K Shih JC. Tissue-specific effects of estrogen on monoamine oxidase A and B in the rat. Life Sci. (1998) 63:155–60. doi: 10.1016/S0024-3205(98)00255-0 9698044

[155] Birzniece V Johansson IM Wang MD Seckl JR Bäckström T Olsson T. Serotonin 5-HT(1A) receptor mRNA expression in dorsal hippocampus and raphe nuclei after gonadal hormone manipulation in female rats. Neuroendocrinology. (2001) 74:135–42. doi: 10.1159/000054679 11474221

[156] Hiroi R Neumaier JF. Estrogen decreases 5-HT1B autoreceptor mRNA in selective subregion of rat dorsal raphe nucleus: Inverse association between gene expression and anxiety behavior in the open field. Neuroscience. (2009) 158:456–64. doi: 10.1016/j.neuroscience.2008.10.016 PMC2667128 19049819

[157] Osterlund MK Overstreet DH Hurd YL. The flinders sensitive line rats, a genetic model of depression, show abnormal serotonin receptor mRNA expression in the brain that is reversed by 17beta-estradiol. Brain Res Mol Brain Res. (1999) 74:158–66. doi: 10.1016/s0169-328x(99)00274-0 10640686

[158] Cyr M Bossé R Di Paolo T. Gonadal hormones modulate 5-hydroxytryptamine2A receptors: emphasis on the rat frontal cortex. Neuroscience. (1998) 83:829–36. doi: 10.1016/S0306-4522(97)00445-4 9483566

[159] Sumner BE Grant KE Rosie R Hegele-Hartung C Fritzemeier KH Fink G. Effects of tamoxifen on serotonin transporter and 5-hydroxytryptamine(2A) receptor binding sites and mRNA levels in the brain of ovariectomized rats with or without acute estradiol replacement. Brain Res Mol Brain Res. (1999) 73:119–28. doi: 10.1016/S0169-328X(99)00243-0 10581405

[160] Osterlund MK Hurd YL. Acute 17 beta-estradiol treatment down-regulates serotonin 5HT1A receptor mRNA expression in the limbic system of female rats. Brain Res Mol Brain Res. (1998) 55:169–72. doi: 10.1016/s0169-328x(98)00018-7 9645972

[161] Mize AL Alper RH. Acute and long-term effects of 17beta-estradiol on G(i/o) coupled neurotransmitter receptor function in the female rat brain as assessed by agonist-stimulated [35S]GTPgammaS binding. Brain Res. (2000) 859:326–33. doi: 10.1016/S0006-8993(00)01998-3 10719081

[162] Mize AL Poisner AM Alper RH. Estrogens act in rat hippocampus and frontal cortex to produce rapid, receptor-mediated decreases in serotonin 5-HT(1A) receptor function. Neuroendocrinology. (2001) 73:166–74. doi: 10.1159/000054633 11307035

[163] McQueen JK Wilson H Fink G. Estradiol-17 beta increases serotonin transporter (SERT) mRNA levels and the density of SERT-binding sites in female rat brain. Brain Res Mol Brain Res. (1997) 45:13–23. doi: 10.1016/S0169-328X(96)00233-1 9105666

[164] Mitra SW Hoskin E Yudkovitz J Pear L Wilkinson HA Hayashi S. Immunolocalization of estrogen receptor beta in the mouse brain: comparison with estrogen receptor alpha [published correction appears in Endocrinology. Endocrinology. (2003) 144:2055–67. doi: 10.1210/en.2002-221069 12697714

[165] Creutz LM Kritzer MF. Estrogen receptor-beta immunoreactivity in the midbrain of adult rats: regional, subregional, and cellular localization in the A10, A9, and A8 dopamine cell groups. J Comp Neurol. (2002) 446:288–300. doi: 10.1002/cne.10207 11932944

[166] Serova L Rivkin M Nakashima A Sabban EL. Estradiol stimulates gene expression of norepinephrine biosynthetic enzymes in rat locus coeruleus. Neuroendocrinology. (2002) 75:193–200. doi: 10.1159/000048237 11914591

[167] Pasqualini C Olivier V Guibert B Frain O Leviel V. Acute stimulatory effect of estradiol on striatal dopamine synthesis. J Neurochem. (1995) 65:1651–7. doi: 10.1046/j.1471-4159.1995.65041651.x 7561861

[168] Etgen AM Karkanias GB. Estrogen regulation of noradrenergic signaling in the hypothalamus. Psychoneuroendocrinology. (1994) 19:603–10. doi: 10.1016/0306-4530(94)90044-2 7938358

[169] Liu B Xie J. Increased dopamine release in *vivo* by estradiol benzoate from the central amygdaloid nucleus of Parkinson's disease model rats. J Neurochem. (2004) 90:654–8. doi: 10.1111/j.1471-4159.2004.02518.x 15255943

[170] Le Saux M Di Paolo T. Influence of oestrogenic compounds on monoamine transporters in rat striatum. J Neuroendocrinol. (2006) 18:25–32. doi: 10.1111/j.1365-2826.2005.01380.x 16451217

[171] Le Saux M Morissette M Di Paolo T. ER beta mediates the estradiol increase of D2 receptors in rat striatum and nucleus accumbens. Neuropharmacology. (2006) 50:451–7. doi: 10.1016/j.neuropharm.2005.10.004 16309717

[172] Jiang H Xie T Ramsden DB Ho SL. Human catechol-O-methyltransferase down-regulation by estradiol. Neuropharmacology. (2003) 45:1011–8. doi: 10.1016/S0028-3908(03)00286-7 14573393

[173] Singh M Meyer EM Simpkins JW. The effect of ovariectomy and estradiol replacement on brain-derived neurotrophic factor messenger ribonucleic acid expression in cortical and hippocampal brain regions of female Sprague-Dawley rats. Endocrinology. (1995) 136:2320–4. doi: 10.1210/endo.136.5.7720680 7720680

[174] Sohrabji F Miranda RC Toran-Allerand CD. Identification of a putative estrogen response element in the gene encoding brain-derived neurotrophic factor. Proc Natl Acad Sci U.S.A. (1995) 92:11110–4. doi: 10.1073/pnas.92.24.11110 PMC40581 7479947

[175] Berchtold NC Kesslak JP Pike CJ Adlard PA Cotman CW. Estrogen and exercise interact to regulate brain-derived neurotrophic factor mRNA and protein expression in the hippocampus. Eur J Neurosci. (2001) 14:1992–2002. doi: 10.1046/j.0953-816x.2001.01825.x 11860494

[176] Blurton-Jones M Kuan PN Tuszynski MH. Anatomical evidence for transsynaptic influences of estrogen on brain-derived neurotrophic factor expression. J Comp Neurol. (2004) 468:347–60. doi: 10.1002/cne.10989 14681930

[177] Blurton-Jones M Tuszynski MH. Estrogen receptor-beta colocalizes extensively with parvalbumin-labeled inhibitory neurons in the cortex, amygdala, basal forebrain, and hippocampal formation of intact and ovariectomized adult rats. J Comp Neurol. (2002) 452:276–87. doi: 10.1002/cne.10393 12353223

[178] Blurton-Jones M Tuszynski MH. Estradiol-induced modulation of estrogen receptor-beta and GABA within the adult neocortex: a potential transsynaptic mechanism for estrogen modulation of BDNF. J Comp Neurol. (2006) 499:603–12. doi: 10.1002/cne.21122 17029253

[179] Hoshaw BA Malberg JE Lucki I. Central administration of IGF-I and BDNF leads to long-lasting antidepressant-like effects. Brain Res. (2005) 1037:204–8. doi: 10.1016/j.brainres.2005.01.007 15777771

[180] El-Bakri NK Islam A Suliman I Lindgren U Winblad B Adem A. Ovariectomy and gonadal hormone treatment: effects on insulin-like growth factor-1 receptors in the rat brain. Growth Horm IGF Res. (2004) 14:388–93. doi: 10.1016/j.ghir.2004.04.004 15336232

[181] Mazzucco CA Lieblich SE Bingham BI Williamson MA Viau V Galea LA. Both estrogen receptor alpha and estrogen receptor beta agonists enhance cell proliferation in the dentate gyrus of adult female rats. Neuroscience. (2006) 141:1793–800. doi: 10.1016/j.neuroscience.2006.05.032 16797852

[182] Barha CK Lieblich SE Galea LA. Different forms of oestrogen rapidly upregulate cell proliferation in the dentate gyrus of adult female rats. J Neuroendocrinol. (2009) 21:155–66. doi: 10.1111/j.1365-2826.2008.01809.x 19076272

[183] Tanapat P Hastings NB Reeves AJ Gould E. Estrogen stimulates a transient increase in the number of new neurons in the dentate gyrus of the adult female rat. J Neurosci. (1999) 19:5792–801. doi: 10.1523/JNEUROSCI.19-14-05792.1999 PMC6783062 10407020

[184] Banasr M Hery M Brezun JM Daszuta A. Serotonin mediates oestrogen stimulation of cell proliferation in the adult dentate gyrus. Eur J Neurosci. (2001) 14:1417–24. doi: 10.1046/j.0953-816x.2001.01763.x 11722603

[185] Aberg MA Aberg ND Hedbäcker H Oscarsson J Eriksson PS. Peripheral infusion of IGF-I selectively induces neurogenesis in the adult rat hippocampus. J Neurosci. (2000) 20:2896–903. doi: 10.1523/JNEUROSCI.20-08-02896.2000 PMC6772218 10751442

[186] Sairanen M Lucas G Ernfors P Castrén M Castrén E. Brain-derived neurotrophic factor and antidepressant drugs have different but coordinated effects on neuronal turnover, proliferation, and survival in the adult dentate gyrus. J Neurosci. (2005) 25:1089–94. doi: 10.1523/JNEUROSCI.3741-04.2005 PMC6725966 15689544

[187] Li Y Luikart BW Birnbaum S Chen J Kwon CH Kernie SG. TrkB regulates hippocampal neurogenesis and governs sensitivity to antidepressive treatment. Neuron. (2008) 59:399–412. doi: 10.1016/j.neuron.2008.06.023 18701066 PMC2655199

[188] Liu F Day M Muñiz LC Bitran D Arias R Revilla-Sanchez R. Activation of estrogen receptor-beta regulates hippocampal synaptic plasticity and improves memory. Nat Neurosci. (2008) 11:334–43. doi: 10.1038/nn2057 18297067

[189] Bethea CL Smith AW Centeno ML Reddy AP. Long-term ovariectomy decreases serotonin neuron number and gene expression in free ranging macaques. Neuroscience. (2011) 192:675–88. doi: 10.1016/j.neuroscience.2011.06.003 PMC3166449 21763405

[190] Hiroi R Handa RJ. Estrogen receptor-β regulates human tryptophan hydroxylase-2 through an estrogen response element in the 5' untranslated region. J Neurochem. (2013) 127:487–95. doi: 10.1111/jnc.12401 PMC5825233 24033289

[191] Metcalf CA Johnson RL Duffy KA Freeman EW Sammel MD Epperson CN. Depressed, stressed, and inflamed: C-reactive protein linked with depression symptoms in midlife women with both childhood and current life stress. Stress Health. (2024) 40:e3313. doi: 10.1002/smi.3313 37679965 PMC10918037

[192] Lund TD Rovis T Chung WC Handa RJ. Novel actions of estrogen receptor-beta on anxiety-related behaviors. Endocrinology. (2005) 146:797–807. doi: 10.1210/en.2004-1158 15514081

[193] Oyola MG Handa RJ. Hypothalamic-pituitary-adrenal and hypothalamic-pituitary-gonadal axes: sex differences in regulation of stress responsivity. Stress. (2017) 20:476–94. doi: 10.1080/10253890.2017.1369523 PMC5815295 28859530

[194] Suzuki H Barros RP Sugiyama N Krishnan V Yaden BC Kim HJ. Involvement of estrogen receptor β in maintenance of serotonergic neurons of the dorsal raphe. Mol Psychiatry. (2013) 18:674–80. doi: 10.1038/mp.2012.62 22665260

[195] Yang F Cheung A Tao J Zhao N Wan W Sheng J. Physiological dosages of estradiol and diarylpropionitrile decrease depressive behavior and increase tryptophan hydroxylase expression in the dorsal raphe nucleus of rats subjected to the forced swim test. Neuroreport. (2019) 30:66–70. doi: 10.1097/WNR.0000000000001158 30379725

[196] Clark MS McDevitt RA Neumaier JF. Quantitative mapping of tryptophan hydroxylase-2, 5-HT1A, 5-HT1B, and serotonin transporter expression across the anteroposterior axis of the rat dorsal and median raphe nuclei. J Comp Neurol. (2006) 498:611–23. doi: 10.1002/cne.21073 16917826

[197] Avis NE Brambilla D McKinlay SM Vass K. A longitudinal analysis of the association between menopause and depression. Results from the Massachusetts Women's Health Study. Ann Epidemiol. (1994) 4:214–20. doi: 10.1016/1047-2797(94)90099-X 8055122

[198] Freeman EW Sammel MD Rinaudo PJ Sheng L. Premenstrual syndrome as a predictor of menopausal symptoms. Obstet Gynecol. (2004) 103:960–6. doi: 10.1097/01.AOG.0000124804.81095.7f 15121571

[199] Hunter MS Gupta P Chedraui P Blümel JE Tserotas K Aguirre W. The international menopause study of climate, altitude, temperature (IMS-CAT) and vasomotor symptoms. Climacteric. (2013) 16:8–16. doi: 10.3109/13697137.2012.699563 22946508

[200] Choi JY Park SJ Lee HJ. Healthy and unhealthy dietary patterns of depressive symptoms in middle-aged women. Nutrients. (2024) 16:776. doi: 10.3390/nu16060776 38542687 PMC10974392

[201] Xu H Liu J Li P Liang Y. Effects of mind-body exercise on perimenopausal and postmenopausal women: a systematic review and meta-analysis. Menopause. (2024) 31:457–67. doi: 10.1097/GME.0000000000002336 PMC11465887 38669625

[202] Afridi I. Psychological and social aspects of menopause. In: Rodríguez-Landa JF Cueto-Escobedo J, editors. A Multidisciplinary Look at Menopause. Croatia: Intech (2017). p. 49–62. doi: 10.5772/intechopen.69078

[203] Bossé R DiPaolo T. The modulation of brain dopamine and GABAA receptors by estradiol: a clue for CNS changes occurring at menopause. Cell Mol Neurobiol. (1996) 16:199–212. doi: 10.1007/BF02088176 8743969 PMC11563143

[204] Monteleone P Mascagni G Giannini A Genazzani AR Simoncini T. Symptoms of menopause - global prevalence, physiology and implications. Nat Endocrinol. (2018) 14:199–215. doi: 10.1038/nrendo.2017.180 29393299

